# Comparative performances of machine learning methods for classifying Crohn Disease patients using genome-wide genotyping data

**DOI:** 10.1038/s41598-019-46649-z

**Published:** 2019-07-17

**Authors:** Alberto Romagnoni, Simon Jégou, Kristel Van Steen, Gilles Wainrib, Jean-Pierre Hugot, International Inflammatory Bowel Disease Genetics Consortium (IIBDGC), Laurent Peyrin-Biroulet, Mathias Chamaillard, Jean-Frederick Colombel, Mario Cottone, Mauro D’Amato, Renata D’Incà, Jonas Halfvarson, Paul Henderson, Amir Karban, Nicholas A. Kennedy, Mohammed Azam Khan, Marc Lémann, Arie Levine, Dunecan Massey, Monica Milla, Sok Meng Evelyn Ng, Ioannis Oikonomou, Harald Peeters, Deborah D. Proctor, Jean-Francois Rahier, Paul Rutgeerts, Frank Seibold, Laura Stronati, Kirstin M. Taylor, Leif Törkvist, Kullak Ublick, Johan Van Limbergen, Andre Van Gossum, Morten H. Vatn, Hu Zhang, Wei Zhang, Jane M. Andrews, Peter A. Bampton, Murray Barclay, Timothy H. Florin, Richard Gearry, Krupa Krishnaprasad, Ian C. Lawrance, Gillian Mahy, Grant W. Montgomery, Graham Radford-Smith, Rebecca L. Roberts, Lisa A. Simms, Katherine Hanigan, Anthony Croft, Leila Amininijad, Isabelle Cleynen, Olivier Dewit, Denis Franchimont, Michel Georges, Debby Laukens, Harald Peeters, Jean-Francois Rahier, Paul Rutgeerts, Emilie Theatre, André Van Gossum, Severine Vermeire, Guy Aumais, Leonard Baidoo, Arthur M. Barrie, Karen Beck, Edmond-Jean Bernard, David G. Binion, Alain Bitton, Steve R. Brant, Judy H. Cho, Albert Cohen, Kenneth Croitoru, Mark J. Daly, Lisa W. Datta, Colette Deslandres, Richard H. Duerr, Debra Dutridge, John Ferguson, Joann Fultz, Philippe Goyette, Gordon R. Greenberg, Talin Haritunians, Gilles Jobin, Seymour Katz, Raymond G. Lahaie, Dermot P. McGovern, Linda Nelson, Sok Meng Ng, Kaida Ning, Ioannis Oikonomou, Pierre Paré, Deborah D. Proctor, Miguel D. Regueiro, John D. Rioux, Elizabeth Ruggiero, L. Philip Schumm, Marc Schwartz, Regan Scott, Yashoda Sharma, Mark S. Silverberg, Denise Spears, A. Hillary Steinhart, Joanne M. Stempak, Jason M. Swoger, Constantina Tsagarelis, Wei Zhang, Clarence Zhang, Hongyu Zhao, Jan Aerts, Tariq Ahmad, Hazel Arbury, Anthony Attwood, Adam Auton, Stephen G. Ball, Anthony J. Balmforth, Chris Barnes, Jeffrey C. Barrett, Inês Barroso, Anne Barton, Amanda J. Bennett, Sanjeev Bhaskar, Katarzyna Blaszczyk, John Bowes, Oliver J. Brand, Peter S. Braund, Francesca Bredin, Gerome Breen, Morris J. Brown, Ian N. Bruce, Jaswinder Bull, Oliver S. Burren, John Burton, Jake Byrnes, Sian Caesar, Niall Cardin, Chris M. Clee, Alison J. Coffey, John MC Connell, Donald F. Conrad, Jason D. Cooper, Anna F. Dominiczak, Kate Downes, Hazel E. Drummond, Darshna Dudakia, Andrew Dunham, Bernadette Ebbs, Diana Eccles, Sarah Edkins, Cathryn Edwards, Anna Elliot, Paul Emery, David M. Evans, Gareth Evans, Steve Eyre, Anne Farmer, I. Nicol Ferrier, Edward Flynn, Alistair Forbes, Liz Forty, Jayne A. Franklyn, Timothy M. Frayling, Rachel M. Freathy, Eleni Giannoulatou, Polly Gibbs, Paul Gilbert, Katherine Gordon-Smith, Emma Gray, Elaine Green, Chris J. Groves, Detelina Grozeva, Rhian Gwilliam, Anita Hall, Naomi Hammond, Matt Hardy, Pile Harrison, Neelam Hassanali, Husam Hebaishi, Sarah Hines, Anne Hinks, Graham A. Hitman, Lynne Hocking, Chris Holmes, Eleanor Howard, Philip Howard, Joanna M. M. Howson, Debbie Hughes, Sarah Hunt, John D. Isaacs, Mahim Jain, Derek P. Jewell, Toby Johnson, Jennifer D. Jolley, Ian R. Jones, Lisa A. Jones, George Kirov, Cordelia F. Langford, Hana Lango-Allen, G. Mark Lathrop, James Lee, Kate L. Lee, Charlie Lees, Kevin Lewis, Cecilia M. Lindgren, Meeta Maisuria-Armer, Julian Maller, John Mansfield, Jonathan L. Marchini, Paul Martin, Dunecan CO Massey, Wendy L. McArdle, Peter McGuffin, Kirsten E. McLay, Gil McVean, Alex Mentzer, Michael L. Mimmack, Ann E. Morgan, Andrew P. Morris, Craig Mowat, Patricia B. Munroe, Simon Myers, William Newman, Elaine R. Nimmo, Michael C. O’Donovan, Abiodun Onipinla, Nigel R. Ovington, Michael J. Owen, Kimmo Palin, Aarno Palotie, Kirstie Parnell, Richard Pearson, David Pernet, John RB Perry, Anne Phillips, Vincent Plagnol, Natalie J. Prescott, Inga Prokopenko, Michael A. Quail, Suzanne Rafelt, Nigel W. Rayner, David M. Reid, Anthony Renwick, Susan M. Ring, Neil Robertson, Samuel Robson, Ellie Russell, David St Clair, Jennifer G. Sambrook, Jeremy D. Sanderson, Stephen J. Sawcer, Helen Schuilenburg, Carol E. Scott, Richard Scott, Sheila Seal, Sue Shaw-Hawkins, Beverley M. Shields, Matthew J. Simmonds, Debbie J. Smyth, Elilan Somaskantharajah, Katarina Spanova, Sophia Steer, Jonathan Stephens, Helen E. Stevens, Kathy Stirrups, Millicent A. Stone, David P. Strachan, Zhan Su, Deborah P. M. Symmons, John R. Thompson, Wendy Thomson, Martin D. Tobin, Mary E. Travers, Clare Turnbull, Damjan Vukcevic, Louise V. Wain, Mark Walker, Neil M. Walker, Chris Wallace, Margaret Warren-Perry, Nicholas A. Watkins, John Webster, Michael N. Weedon, Anthony G. Wilson, Matthew Woodburn, B. Paul Wordsworth, Chris Yau, Allan H. Young, Eleftheria Zeggini, Matthew A. Brown, Paul R. Burton, Mark J. Caulfield, Alastair Compston, Martin Farrall, Stephen C. L. Gough, Alistair S. Hall, Andrew T. Hattersley, Adrian V. S. Hill, Christopher G. Mathew, Marcus Pembrey, Jack Satsangi, Michael R. Stratton, Jane Worthington, Matthew E. Hurles, Audrey Duncanson, Willem H. Ouwehand, Miles Parkes, Nazneen Rahman, John A. Todd, Nilesh J. Samani, Dominic P. Kwiatkowski, Mark I. McCarthy, Nick Craddock, Panos Deloukas, Peter Donnelly, Jenefer M. Blackwell, Elvira Bramon, Juan P. Casas, Aiden Corvin, Janusz Jankowski, Hugh S. Markus, Colin NA Palmer, Robert Plomin, Anna Rautanen, Richard C. Trembath, Ananth C. Viswanathan, Nicholas W. Wood, Chris C. A. Spencer, Gavin Band, Céline Bellenguez, Colin Freeman, Garrett Hellenthal, Eleni Giannoulatou, Matti Pirinen, Richard Pearson, Amy Strange, Hannah Blackburn, Suzannah J. Bumpstead, Serge Dronov, Matthew Gillman, Alagurevathi Jayakumar, Owen T. McCann, Jennifer Liddle, Simon C. Potter, Radhi Ravindrarajah, Michelle Ricketts, Matthew Waller, Paul Weston, Sara Widaa, Pamela Whittaker

**Affiliations:** 1 Centre de recherche sur l’inflammation UMR 1149, Inserm - Université Paris Diderot, 75018 Paris, France; 2 Data Team, Département d’informatique de l’ENS, École normale supérieure, CNRS, PSL Research University, 75005 Paris, France; 3 Owkin, 75011 Paris, France; 4 WELBIO, GIGA-R Medical Genomics - BIO3, University of Liège, Liège, Belgium; 5 Department of Human Genetics, University of Leuven, Leuven, Belgium; 6 Hôpital Robert Debré, Assistance Publique-Hôpitaux de Paris, 75019 Paris, France; 7 Gastroenterology Unit, INSERM U954, Nancy University and Hospital, Nancy, France; 8 INSERM, U1019 Lille, France; 9 Univ Lille Nord de France, CHU Lille and Lille-2 University, Gastroenterology Unit, Lille, France; 10 Division of Internal Medicine, Villa Sofia-V. Cervello Hospital, University of Palermo, Palermo, Italy; 11 Department of Biosciences and Nutrition, Karolinska Institutet, Stockholm, Sweden; 12 Department of Surgical and Gastroenterological Sciences, University of Padua, Padua, Italy; 13 Department of Medicine, Örebro University Hospital, Örebro, Sweden; 14 School of Health and Medical Sciences, Örebro University, Örebro, Sweden; 15 Royal Hospital for Sick Children, Paediatric Gastroenterology and Nutrition, Edinburgh, UK; 16 Child Life and Health, University of Edinburgh, Edinburgh, UK; 17 Department of Gastroenterology, Faculty of Medicine, Technion- Israel Institute of Technology, Haifa, Israel; 18 Gastrointestinal Unit, Institute of Genetics and Molecular Medicine, University of Edinburgh, Edinburgh, UK; 19 Genetic Medicine, MAHSC, University of Manchester, Manchester, UK; 20 Université Paris Diderot, GETAID group, Paris, France; 21 Pediatric Gastroenterology Unit, Wolfson Medical Center and Sackler School of Medicine, Tel Aviv University, Tel Aviv, Israel; 22 Inflammatory Bowel Disease Research Group, Addenbrooke’s Hospital, University of Cambridge, Cambridge, UK; 23 Azienda Ospedaliero Universitaria (AOU) Careggi, Unit of Gastroenterology, SOD2 Florence, Italy; 24 Department of Internal Medicine, Section of Digestive Diseases, Yale School of Medicine, New Haven, Connecticut USA; 25 Dept Gastroenterology - University hospital Gent - De Pintelaan - 9000, Gent, Belgium; 26 Dept Gastroenterology - UCL Mont Godinne, Namur, Belgium; 27 Division of Gastroenterology, University Hospital Gasthuisberg, Leuven, Belgium; 28 University of Bern, Division of Gastroenterology, Inselspital, Bern, Switzerland; 29 Department of Radiobiology and Human Health, Italian National Agency for New Technologies, Energy and Sustainable Economic Development (ENEA), Rome, Italy; 30 Dept Gastroenterology, Guy’s & St. Thomas’ NHS Foundation Trust, St Thomas’ Hospital, London, UK; 31 Department of Clinical Science, Intervention and Technology, Karolinska Institutet, Stockholm, Sweden; 32 Division of Clinical Pharmacology and Toxicology, University Hospital Zurich, Zurich, Switzerland; 33 Division of Pediatric Gastroenterology, Hepatology and Nutrition, Hospital for Sick Children, Toronto, Ontario, Canada; 34 Dept Gastroenterology - 3University Brussels, Brussels, Belgium; 35 Department of Transplantation Medicine, Division of Cancer medicine, Surgery and Transplantation, Oslo University Hospital Rikshospitalet, Oslo, Norway; 36 Inflammatory Bowel Disease Service, Department of Gastroenterology and Hepatology, Royal Adelaide Hospital, and School of Medicine, University of Adelaide, Adelaide, Australia; 37 Department of Gastroenterology and Hepatology, Flinders Medical Centre and School of Medicine, Flinders University, Adelaide, Australia; 38 Department of Gastroenterology, Christchurch Hospital and Department of Medicine, University of Otago, Christchurch, New Zealand; 39 Department of Gastroenterology, Mater Health Services, Brisbane, Australia; 40 School of Medicine, University of Queensland, Brisbane, Australia; 41 Inflammatory Bowel Diseases, Genetics and Computational Biology, Queensland Institute of Medical Research, Brisbane, Australia; 42 Centre for Inflammatory Bowel Diseases, Fremantle Hospital and School of Medicine and Pharmacology, The University of Western Australia, Fremantle, Australia; 43 Department of Gastroenterology, The Townsville Hospital and James Cook University School of Medicine, Townsville, Australia; 44 Molecular Epidemiology, Genetics and Computational Biology, Queensland Institute of Medical Research, Brisbane, Australia; 45 Department of Gastroenterology, Royal Brisbane and Womens Hospital, and School of Medicine, University of Queensland, Brisbane, Australia; 46 University of Otago, Department of Medicine, Christchurch, New Zealand; 47 Erasmus Hospital, Free University of Brussels, Department of Gastroenterology, Brussels, Belgium; 48 Department of Pathophysiology, Gastroenterology section, KU Leuven, Leuven, Belgium; 49 Department of Gastroenterology, Clinique Universitaire St-Luc, Brussels, Belgium; 50 Unit of Animal Genomics, Groupe Interdisciplinaire de Gnoprotomique Applique (GIGA-R) and Faculty of Veterinary Medicine, University of Lige, Lige, Belgium; 51 Ghent University Hospital, Department of Gastroenterology and Hepatology, Ghent, Belgium; 52 Division of Gastroenterology, Centre Hospitalier Universitaire, Universit de Lige, Lige, Belgium; 53 Division of Gastroenterology, University Hospital Gasthuisberg, Leuven, Belgium; 54 University of Montreal, Maissonneuve’ Rosemont Hospital, Quebec Association of Gastroenterologists, Montréal, Québec, Canada; 55 Division of Gastroenterology, Hepatology and Nutrition, Department of Medicine, University of Pittsburgh School of Medicine, Pittsburgh, Pennsylvania USA; 56 Hôpital Hôtel Dieu, Montréal, Québec, Canada; 57 Division of Gastroenterology, McGill University Health Centre, Royal Victoria Hospital, Montréal, Québec Canada; 58 Inflammatory Bowel Disease Center, Department of Medicine, Johns Hopkins University School of Medicine, Baltimore, Maryland USA; 59 Department of Genetics, Yale School of Medicine, New Haven, Connecticut USA; 60 Department of Internal Medicine, Section of Digestive Diseases, Yale School of Medicine, New Haven, Connecticut USA; 61 Division of Gastroenterology, Hôpital Général Juif Sir Mortimer B. Davis Jewish General Hospital, Montréal, Québec, Canada; 62 Mount Sinai Hospital Inflammatory Bowel Disease Centre, University of Toronto, Toronto, Ontario Canada; 63 Analytic and Translational Genetics Unit, Massachusetts General Hospital, Harvard Medical School, Boston, Massachusetts USA; 64 Broad Institute of MIT and Harvard, Cambridge, Massachusetts USA; 65 Hopital Sainte Justine, Montréal, Québec Canada; 66 Department of Human Genetics, University of Pittsburgh Graduate School of Public Health, Pittsburgh, Pennsylvania USA; 67 Medical Genetics Institute, Cedars-Sinai Medical Center, Los Angeles, California USA; 68 Université de Montréal and the Montreal Heart Institute, Research Center, Montréal, Québec Canada; 69 Pavillon Maisonneuve, Montréal, Québec Canada; 70 Long Island Clinical Research Associates, Great Neck, New York USA; 71 CHUM’ Hopital Sainte-Luc, Montréal, Québec Canada; 72 Inflammatory Bowel and Immunobiology Research Institute, Cedars-Sinai Medical Center, Los Angeles, California USA; 73 Laval University, Quebec City, Québec, Canada; 74 Department of Health Studies, University of Chicago, Chicago, Illinois USA; 75 Department of Biostatistics, School of Public Health, Yale University, New Haven, Connecticut USA; 76 The Wellcome Trust Sanger Institute, Wellcome Trust Genome Campus, Hinxton, Cambridge CB10 1SA UK; 77 Genetics of Complex Traits, Peninsula College of Medicine and Dentistry University of Exeter, Exeter, EX1 2LU UK; 78 Department of Haematology, University of Cambridge, Long Road, Cambridge, CB2 0PT UK; 79 National Health Service Blood and Transplant, Cambridge Centre, Long Road, Cambridge, CB2 0PT UK; 80 Department of Statistics, University of Oxford, 1 South Parks Road, Oxford, OX1 3TG UK; 81 Multidisciplinary Cardiovascular Research Centre (MCRC), Leeds Institute of Genetics, Health and Therapeutics (LIGHT), University of Leeds, Leeds, LS2 9JT UK; 82 ARC Epidemiology Unit, Stopford Building, University of Manchester, Oxford Road, Manchester, M13 9PT UK; 83 Oxford Centre for Diabetes, Endocrinology and Medicine, University of Oxford, Churchill Hospital, Oxford, OX3 7LJ UK; 84 Department of Medical and Molecular Genetics, King’s College London School of Medicine, 8th Floor Guy’s Tower, Guy’s Hospital, London, SE1 9RT UK; 85 Centre for Endocrinology, Diabetes and Metabolism, Institute of Biomedical Research, University of Birmingham, Birmingham, B15 2TT UK; 86 Department of Cardiovascular Sciences, University of Leicester, Glenfield Hospital, Groby Road, Leicester, LE3 9QP UK; 87 IBD Genetics Research Group, Addenbrooke’s Hospital, Cambridge, CB2 0QQ UK; 88 University of Aberdeen, Institute of Medical Sciences, Foresterhill, Aberdeen AB25 2ZD UK; 89 SGDP, The Institute of Psychiatry, King’s College London, De Crespigny Park, Denmark Hill, London SE5 8AF UK; 90 Clinical Pharmacology Unit, University of Cambridge, Addenbrookes Hospital, Hills Road, Cambridge, CB2 2QQ UK; 91 Section of Cancer Genetics, Institute of Cancer Research, 15 Cotswold Road, Sutton, SM2 5NG UK; 92 Juvenile Diabetes Research Foundation/Wellcome Trust Diabetes and Inflammation Laboratory, Department of Medical Genetics, Cambridge Institute for Medical Research, University of Cambridge, Wellcome Trust/MRC Building, Cambridge, CB2 0XY UK; 93 The Wellcome Trust Centre for Human Genetics, University of Oxford, Roosevelt Drive, Oxford, OX3 7BN UK; 94 Department of Psychiatry, University of Birmingham, National Centre for Mental Health, 25 Vincent Drive, Birmingham, B15 2FG UK; 95 BHF Glasgow Cardiovascular Research Centre, University of Glasgow, 126 University Place, Glasgow, G12 8TA UK; 96 Gastrointestinal Unit, Division of Medical Sciences, School of Molecular and Clinical Medicine, University of Edinburgh, Western General Hospital, Edinburgh, EH4 2XU UK; 97 Academic Unit of Genetic Medicine, University of Southampton, Southampton, UK; 98 Endoscopy Regional Training Unit, Torbay Hospital, Torbay, TQ2 7AA UK; 99 Academic Unit of Musculoskeletal Disease, University of Leeds, Chapel Allerton Hospital, Leeds, West Yorkshire LS7 4SA UK; 100 MRC Centre for Causal Analyses in Translational Epidemiology, Department of Social Medicine, University of Bristol, Bristol, BS8 2BN UK; 101 Department of Medical Genetics, Manchester Academic Health Science Centre (MAHSC), University of Manchester, Manchester, M13 0JH UK; 102 School of Neurology, Neurobiology and Psychiatry, Royal Victoria Infirmary, Queen Victoria Road, Newcastle upon Tyne, NE1 4LP UK; 103 Institute for Digestive Diseases, University College London Hospitals Trust, London, NW1 2BU UK; 104 MRC Centre for Neuropsychiatric Genetics and Genomics, School of Medicine, Cardiff University, Heath Park, Cardiff, CF14 4XN UK; 105 University Hospital Birmingham NHS Foundation Trust, Birmingham, B15 2TT UK; 106 University of Oxford, Institute of Musculoskeletal Sciences, Botnar Research Centre, Oxford, OX3 7LD UK; 107 Centre for Diabetes and Metabolic Medicine, Barts and The London, Royal London Hospital, Whitechapel, London E1 1BB UK; 108 Bone Research Group, Department of Medicine and Therapeutics, University of Aberdeen, Aberdeen, AB25 2ZD UK; 109 Clinical Pharmacology and Barts and The London Genome Centre, William Harvey Research Institute, Barts and The London School of Medicine and Dentistry, Queen Mary University of London, Charterhouse Square, London, EC1M 6BQ UK; 110 Institute of Cellular Medicine, Musculoskeletal Research Group, 4th Floor, Catherine Cookson Building, The Medical School, Framlington Place, Newcastle upon Tyne, NE2 4HH UK; 111 Gastroenterology Unit, Radcliffe Infirmary, University of Oxford, Oxford, OX2 6HE UK; 112 Centre National de Genotypage, 2, Rue Gaston Cremieux, Evry, Paris 91057 France; 113 Department of Gastroenterology & Hepatology, University of Newcastle upon Tyne, Royal Victoria Infirmary, Newcastle upon Tyne, NE1 4LP UK; 114 ALSPAC Laboratory, Department of Social Medicine, University of Bristol, Bristol, BS8 2BN UK; 115 Division of Nutritional Sciences, King’s College London School of Biomedical and Health Sciences, London, SE1 9NH UK; 116 NIHR-Leeds Musculoskeletal Biomedical Research Unit, University of Leeds, Chapel Allerton Hospital, Leeds, West Yorkshire LS74SA UK; 117 Department of General Internal Medicine, Ninewells Hospital and Medical School, Ninewells Avenue, Dundee, DD1 9SY UK; 118 Department of Clinical Neurosciences, University of Cambridge, Addenbrooke’s Hospital, Hills Road, Cambridge, CB2 2QQ UK; 119 Clinical and Academic Rheumatology, Kings College Hosptal National Health Service Foundation Trust, Denmark Hill, London, SE5 9RS UK; 120 University of Toronto, St. Michael’s Hospital, 30 Bond Street, Toronto, Ontario M5B 1W8 Canada; 121 University of Bath, Claverdon, Norwood House, Room 5.11a, Bath Somerset, BA2 7AY UK; 122 Division of Community Health Sciences, St George’s, University of London, London, SW17 0RE UK; 123 Departments of Health Sciences and Genetics, University of Leicester, 217 Adrian Building, University Road, Leicester, LE1 7RH UK; 124 Diabetes Research Group, School of Clinical Medical Sciences, Newcastle University, Framlington Place, Newcastle upon Tyne, NE2 4HH UK; 125 Medicine and Therapeutics, Aberdeen Royal Infirmary, Foresterhill, Aberdeen, Grampian AB9 2ZB UK; 126 School of Medicine and Biomedical Sciences, University of Sheffield, Sheffield, S10 2JF UK; 127 Nuffield Department of Orthopaedics, Rheumatology and Musculoskeletal Sciences, Nuffield Orthopaedic Centre, University of Oxford, Windmill Road, Headington, Oxford, OX3 7LD UK; 128 UBC Institute of Mental Health, 430-5950 University Boulevard Vancouver, British Columbia, V6T 1Z3 Canada; 129 Diamantina Institute of Cancer, Immunology and Metabolic Medicine, Princess Alexandra Hospital, University of Queensland, Ipswich Road, Woolloongabba, Brisbane, Queensland 4102 Australia; 130 Cardiovascular Medicine, University of Oxford, Wellcome Trust Centre for Human Genetics, Roosevelt Drive, Oxford, OX3 7BN UK; 131 Genetics of Diabetes, Peninsula College of Medicine and Dentistry, University of Exeter, Barrack Road, Exeter, EX2 5DW UK; 132 Clinical and Molecular Genetics Unit, Institute of Child Health, University College London, 30 Guilford Street, London, WC1N 1EH UK; 133 The Wellcome Trust, Gibbs Building, 215 Euston Road, London, NW1 2BE UK; 134 Leicester NIHR Biomedical Research Unit in Cardiovascular Disease, Glenfield Hospital, Leicester, LE3 9QP UK; 135 Oxford NIHR Biomedical Research Centre, Churchill Hospital, Oxford, OX3 7LJ UK; 136 Telethon Institute for Child Health Research, Centre for Child Health Research, University of Western Australia, 100 Roberts Road, Subiaco, Western Australia 6008 Australia; 137 Cambridge Institute for Medical Research, University of Cambridge School of Clinical Medicine, Cambridge, CB2 0XY UK; 138 Department of Psychosis Studies, NIHR Biomedical Research Centre for Mental Health at the Institute of Psychiatry, King’s College London and The South London and Maudsley NHS Foundation Trust, Denmark Hill, London, SE5 8AF UK; 139 Department Epidemiology and Population Health, London School of Hygiene and Tropical Medicine, London, WC1E 7HT UK; 140 Dept Epidemiology and Public Health, University College London, London, WC1E 6BT UK; 141 Neuropsychiatric Genetics Research Group, Institute of Molecular Medicine, Trinity College Dublin, Dublin 2, Eire UK; 142 Department of Oncology, Old Road Campus, University of Oxford, Oxford, OX3 7DQ UK; 143 Digestive Diseases Centre, Leicester Royal Infirmary, Leicester, LE7 7HH UK; 144 Centre for Digestive Diseases, Queen Mary University of London, London, E1 2AD UK; 145 Clinical Neurosciences, St George’s University of London, London, SW17 0RE UK; 146 Biomedical Research Centre, Ninewells Hospital and Medical School, Dundee, DD1 9SY UK; 147 NIHR Biomedical Research Centre for Ophthalmology, Moorfields Eye Hospital NHS Foundation Trust and UCL Institute of Ophthalmology, London, EC1V 2PD UK; 148 Department Molecular Neuroscience, Institute of Neurology, Queen Square, London, WC1N 3BG UK

**Keywords:** Crohn's disease, Genetics research

## Abstract

Crohn Disease (CD) is a complex genetic disorder for which more than 140 genes have been identified using genome wide association studies (GWAS). However, the genetic architecture of the trait remains largely unknown. The recent development of machine learning (ML) approaches incited us to apply them to classify healthy and diseased people according to their genomic information. The Immunochip dataset containing 18,227 CD patients and 34,050 healthy controls enrolled and genotyped by the international Inflammatory Bowel Disease genetic consortium (IIBDGC) has been re-analyzed using a set of ML methods: penalized logistic regression (LR), gradient boosted trees (GBT) and artificial neural networks (NN). The main score used to compare the methods was the Area Under the ROC Curve (AUC) statistics. The impact of quality control (QC), imputing and coding methods on LR results showed that QC methods and imputation of missing genotypes may artificially increase the scores. At the opposite, neither the patient/control ratio nor marker preselection or coding strategies significantly affected the results. LR methods, including Lasso, Ridge and ElasticNet provided similar results with a maximum AUC of 0.80. GBT methods like XGBoost, LightGBM and CatBoost, together with dense NN with one or more hidden layers, provided similar AUC values, suggesting limited epistatic effects in the genetic architecture of the trait. ML methods detected near all the genetic variants previously identified by GWAS among the best predictors plus additional predictors with lower effects. The robustness and complementarity of the different methods are also studied. Compared to LR, non-linear models such as GBT or NN may provide robust complementary approaches to identify and classify genetic markers.

## Introduction

Crohn Disease (CD) is an inflammatory bowel disease (IBD) characterized by a chronic or relapsing inflammation of the gut with a prevalence of at least 0.1% in most developed countries^1^. It has been extensively studied by several groups, often in the context of the International IBD Genetics Consortium (IIBDGC), thus sharing common datasets and allowing comparisons between different approaches.

CD is a complex genetic disorder caused by multiple genetic and environmental factors. A major goal of medical genetics is to accurately predict CD from these genetic and environmental parameters. In practice, we need to know risk factors, their effect sizes and how they interact. Environmental risk factors remain largely unknown, except cigarette smoking which is associated with a two-fold increased risk. In comparison, many common polymorphisms that are associated with IBD risk in the population have been identified up to date. Now, the question is to know how they can be used to predict an individual’s genetic risk. “Genetic architecture of a disease refers to the number of genetic polymorphisms that affect the disease risk, the distribution of their allelic frequencies, the distribution of their effect sizes and their genetic mode of action (additive, dominant and/or epistatic)^2^”. More than 200 IBD associated loci have been recognized up to date^3,4^. Except for special clinical situations (like very early onset IBD), rare alleles with large effects (Odds Ratio (OR) > 2) have rarely been detected despite high throughout sequencing methods applied to a large number of patients^5,6^. Most associated variants are common with minor allele frequency (MAF) > 0:01) and risk alleles are either the minor or the major alleles. Their effect sizes are usually small (OR < 1:5) and often smaller (from 1:1 and to 1:2). For most of the identified polymorphisms, the genetic mode of action is multiplicative on the risk scale. Some SNPs have been specifically associated to CD in smokers^7^. However, despite this huge knowledge, the genetic architecture of the traits remains largely unknown as supported by the fact that we explain today only 13% of the genetic variance deduced from twin and family studies^3,4^.

Until now the genomic information has mainly been exploited on the basis of single-locus statistical analyses. However, this approach is under-powered to detect variants carrying low marginal effects alone but strong effects in association with other ones. The phenomenon that the effect of one variant depends on additional variants elsewhere in the genome is known as epistasis or genetic interaction. In case of strong genetic interaction, because only a combination of variants allows predicting the individual risk, the goal of geneticists is to find a risk equation combining presence/absence of each genetic variant to provide personalized predictions. Detecting genetic interaction would also greatly improve the explained genetic variance. However, optimal methods for selecting and combining SNPs remain to be developed.

Several methods have been proposed to analyze whole genotyping datasets looking for genetic interactions (for a review and their application to CD see^8,9^). A search for specific pairwise gene-gene interactions of known genetic factors was initially performed using statistical methods but it failed to identify genetic variants with strong interactions^10,11^. Genome-wide scans looking for two-loci interactions also failed to identify statistically significant epistatic effects^8,12^. Standard methods applied to higher orders of interactions were quickly limited by the multi-testing issue, the size of the datasets and computing power. For these reasons, more sophisticated machine learning (ML) methods have been proposed in order to capture the whole information of GWAS datasets using a direct pan-genomic approach. To explore the performances of different methods, the receiver operator characteristic (ROC) curve and its maximum Area Under Curve (AUC) are often used to compare the sensitivity and specificity of genetic tests in correctly classifying affected and unaffected individuals^2^.

The prediction of a specific phenotype such as “CD” or “non-CD” from raw genomic data such as SNPs can be thought in the framework of supervised learning as a binary classification problem. During training, the algorithm learns from a genotyping dataset and adjusts its internal parameters to minimize an error cost function between predicted probabilities of phenotypes and actual patients phenotypes. After training, the performance of the system is measured on another set of a completely new case/control sample, to evaluate the generalization ability of the proposed algorithm.

Linear ML methods including multivariate logistic regression (LR) and sparse penalized methods such as Lasso have been proposed to identify disease associated SNPs^13^. Penalized methods perform two main tasks in the same process. First, it identifies a set of SNPs involved in disease prediction. Second, it calculates a weight for each SNP which reflects its contribution to the general model. Using the Welcome Trust GWAS dataset, Abraham *et al*. showed that penalized models achieved better performance than non-penalized methods even if the maximum AUC was no more than 0.76^13^. Using either GWAS datasets and/or Immunochip datasets, Chen *et al*. found an AUC of 0.80^14^. Wei *et al*. applied a LR with L1 penalty to the Immunochip dataset and obtained an AUC of 0.86^15^. The observed differences are likely explained by the different datasets used and the quality controls (QC) applied^13,14^.

Penalized or non penalized LR are not suited to capture non linear interactions between loci because they only capture additive risk contributions. A non-linear model achieving better disease prediction results (in term of AUC score in our case), would allow to evaluate the amount of extra-information related to such interactions and provide some hint about their nature. To explore non linear interactions, ensemble tree-based methods such as random forest and gradient boosted trees (GBT) have been proposed^8,16,17^. In these methods, which are often providing state-of-the art results for structured datasets, ensembles of decision trees are trained to minimize the prediction error. Single trees, random forest and Bayesian models have been applied to CD with reported AUC in the same range or lower than penalized LR methods^8,15,18–20^.

Besides ensemble tree-based methods, other tools can be used to capture non-linear interactions. In particular, artificial neural networks (NN) are a powerful class of algorithms to learn non-linear relationships between an input - here the genotype - and a target variable - here the CD phenotype. Despite a long history, this class of algorithms has recently emerged in the framework of deep learning as a state-of-the-art solution to solve major artificial intelligence problems such as image classification, speech recognition or text translation^21^, relying on “signal-type” unstructured data.

Compared to tree-based methods, NN enable to build hierarchical internal representations of the data. Each neuron treats the information from several inputs (like the presence or not of a specific allele) and produces an output which is itself used by another neuron in the following layer. This structure draws multiple levels of representation. Starting from the raw inputs, it forms non-linear combinations of the initial features, each one providing a new representation of the initial data. By adding deeper layers, the networks build higher levels of abstraction and complexity which could be interpreted as biological functions. Deep network are known to amplify relevant inputs and lower the noise in data like images and sounds. However, the application of these approaches to standard tabular datasets does not generally outperform ensemble tree-based methods and it remains an open challenge to design efficient deep learning systems for this type of data. Despite its ability to explore intricate structures in high-dimensional data^22,23^, to our knowledge it has rarely been applied to population genetics and GWAS datasets^24^.

IIBDGC has collected a large dataset from CD patients and healthy controls genotyped for more than 150 thousands genetic variants - mainly single nucleotide polymorphisms (SNPs)- forming the Immunochip panel^3,25^. Using this dataset, we first explored the impact of QC methods, allele coding strategies and marker selections on the test accuracy of Lasso as the reference method. Second, we applied a panel of different penalized logistic regression, GBT and NN methods. Finally, we explored the robustness of the three approaches and their complementarity.

## Methods

All methods have been carried out in accordance with relevant guidelines and regulations. All participants gave a written informed consent. The study has been approved by all the relevant national ethic committees as previously reported^3^.

### Data pre-processing

The original cohort consisted in a total of 51951 people of mainly European descent (22208 males and 30069 females), divided as 18227 Crohn disease (CD) cases and 34050 controls. DNA samples were genotyped for the set of autosomal variants defined in the custom Illumina Infinium chip^25^. Genetic variants consisted in biallelic SNPs and a few small insertion deletions polymorphisms^25^. 156499 variants survived after a first QC performed according to the international consortium^3^. The density of the variants was not uniform along the genome, as previously reported^14^ (Fig. S1), genetic variants previously associated to immune disorders being over-represented^25^.

In the following we call **A** the major allele and **a** the minor one at a given site. Due to the biallelic nature of SNPs under study, in the dataset we substituted them by numerical values 0 and 1, and call *N*_*SNP*_ the number of SNPs.

#### Quality control and imputation

It is well known that QC and imputation on GWAS data are delicate pre-processing steps for any genotype-phenotype association analysis, and that they can strongly affect results and biological interpretation^26–28^. Since one of the aim of this paper is to compare different ML approaches to the classification case-control problem, we first addressed the question of the impact of such an issue, on the AUC scores and on the interpretability of the feature importance selection.

In particular, for this part of the analysis, we have considered two cases:

NoQC - All samples and SNPs are kept for the analysis.

QC - The IIBDGC dataset is pre-processed by applying the cuts on samples and SNPs missing rates as in^3^. In particular, after excluding samples with missing SNP rate greater than 5%, SNPs with sample missing rate greater than 2% and with Hardy-Weinberg equilibrium (HWE) p-value < 10^−10^ in controls, we are left with 17966 CD cases, 33985 controls, for 146237 SNPs.

Moreover we proposed three different schemes to treat unknown genotypes in the dataset (notice that more sophisticated algorithms devoted to imputation, like for example IMPUTE2^29^, could in principle perform better. However, in this work we focused on comparing different ML algorithms on a given unbiased dataset. As we show in the Results Section, strategy B3 is sufficient to this aim). For any given allele in the SNP we:

Unkw - leave the unknown values and treat them as a separate allele, or

Maj - substitute them with the most common allele, or

HW - substitute them with a random choice, following a binomial distribution that satisfy the Hardy-Weinberg equilibrium, for the controls (HW_*c*_), or for all samples (HW_*a*_).

#### Coding

The dataset is unphased, namely alleles in each SNP cannot be ordered for a given sample (phase information does not seem to strongly improve the results in similar setups^30^). Therefore only 3 classes can be associated to each SNP. Different models can be considered, depending on the assumptions on these 3 classes. In the additive model, the SNP genotypes are ordered numbers: without loss of generality one can fix **AA** = 0, **Aa** = 1, **aa** = 2, implying that each additional number of copies of the minor allele increases the risk by the same amount. This coding method is referred as *sum*. A dominant model compares **AA** versus **Aa** + **aa**, and a recessive model compares **AA** + **Aa** versus **aa**, giving rise to only two effective classes (0 and 1). Finally, the three classes can be considered independently, if no strong assumptions can be made about dominance or additivity. This can be achieved in two different ways. The first option is to use a One-Hot Encoding on the three classes, giving rise to an effective number of 3_*_N_*SNP*_ features in the dataset. We refer to this coding as the *OHE* coding. The second possibility is to keep the data as in its raw version (then called *raw* coding in the following), meaning considering each allele as an independent feature. It is easy to convince themselves that this is equivalent to consider the 3 independent classes (0-0, 0-1, 1-1). The advantage of this second option with respect to the OHE model is that the number of effective features is now 2∗*N*_*SNP*_. In this paper, we focus on *sum*, *OHE* and *raw* coding. When interested to a not imputed dataset (referred above as Unkw), only categorical features make sense and OHE coding. Since in the Immunochip dataset the alleles for a given SNP are always either both known or both unknown, in this case one can code only the 4 classes 0-0, 0-1, 1-1, U-U (where U is an unknown allele).

#### Data separation, score, cross-validation

Samples have been randomly permuted, in order to obtain a similar case/control ratio on all subsets. Then the dataset has been separated in a Train dataset (34634 samples) and a Test dataset (17317 samples). In both Train and Test sets, the case/control ratio was around 0.53. We used the area under the ROC curve (AUC) as the score to evaluate the predictions of the different models. For all the models under study, to avoid over-fitting, we optimized the corresponding hyper-parameters by a 10-fold cross-validation on the Train set (see details in the Supp. Info. text). We then evaluated on the Test set the models trained on the entire Train set.

#### SNPs preselection

Association analyses by comparing allele or genotype frequencies between cases and controls are widely used for GWAS. The most commonly used approach is indeed the single SNP scan, consisting in testing each SNP sequentially with the null hypothesis of no association.

Different tests can be used in order to associate SNPs to the phenotype. For example, the additive genetic model can be tested using the Cochran-Armitage trend test^31–33^, which is equivalent to the score test in the LR^34^. Nevertheless, different contingency matrices and corresponding chi-squared test can be studied, depending on the genetic model taken into account.

ML algorithms can in principle deal with genome-wide SNPs. However, dataset with a large number of features are subject to the curse of dimensionality. Therefore, a more efficient strategy consists in first reducing the total number of SNPs to a manageable level via a screening procedure, and look for causal loci among those passing a given threshold^15,35,36^.

In^15^ for example, a SNP preselection based on minimal allele frequency (MAF) threshold and p-values on single SNP association tests, is followed by a LR with Lasso regularization. Since in this case the aim is just to reduce the dimensionality of the space in which the data are embedded, less stringent thresholds than in association studies are used. In this paper we used the threshold values ${\rm{MAF}} > 0.01$ and $p < {10}^{-4}$, considered in^15^, as a benchmark point, and we studied the effects of changing them on the AUC score and on the feature selection at the level of SNPs and loci.

Moreover, in order to be as independent as possible from the assumptions of genetic models when selecting the panel of retained SNPs, we combined the results of different preselection methods. Namely, we kept the union of the SNP lists arising from chi-squared tests based on contingency matrices built on the independent classes model, on the dominant model, on a Cochran-Armitage trend test and on combined alleles counting. In the benchmark case, 120636 SNPs have MAF above the threshold, while 18381, 18200, 19176, and 20716 SNPs passed respectively the cut-off p-values of 10^−4^ for the tests mentioned above. Of those, 14606 lie in the intersections of these lists, while 21896 in the union, which is the final preselection we kept for this benchmark dataset.

### Models and implementation

**Figure 1:**
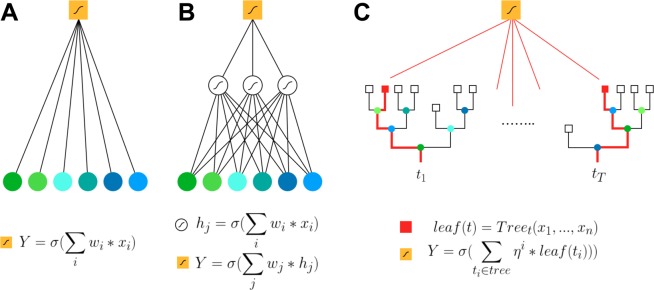
Schematic representation of the machine learning models used in this paper for case/control classification. Colored circles represent input variables *x*_*i*_, yellow square the output prediction *Y*. Small wave symbol represents a sigmoidal function, used to transform a quantitative parameter into a probability of disease association. All formula are approximated and meant to give an idea of the models. (**A**) Logistic Regression: the prediction of this model is given by applying a sigmoid function to a weighted sum of the inputs. (**B**) Dense Neural Networks: they can be seen as multiple stacked logistic regressions. Here we represent a simplified network with one hidden layer with 3 neurons, and the output layer with 1 neuron. Each neuron receives the sigmoid of the weighted sum of its inputs. (**C**) Gradient Boosting on Decision Trees: the prediction is given by the sigmoid of the sum of the outputs leafs of hundreds of decision trees ($\eta$ being the learning rate).

In this paper we considered three classes of models for case/control classification: logistic regression (LR), dense neural networks (NN) and gradient boosting on decision trees (GBT). Figure 1 shows a sketch of the different models strategies to associate a probability to a collection of input variables, in our case the probability of developing the disease starting from genotype data. All data and model analyses have been performed in Python, with an extensive use of the library Scikit-learn^37^. All details concerning the parameters used in the different models can be found in the Supplementary Information text.

#### Logistic regression

In binary classification problems, LR is often the most natural choice to associate to each sample a probability to belong to one of the two classes. It is a particularly powerful model in the cases in which the log-odds of these probabilities only depend on a linear combination of the original features. Penalized (or regularized) LR imposes penalty terms to the logistic model in order to avoid the overfitting problem. In our analysis we considered Lasso (L1), Ridge (L2) and ElasticNet (combined L1 and L2) regularizations. All LR models have been implemented using Scikit-learn^37^.

#### Neural networks

Feed forward fully connected (dense) NN are a family of non-linear models that share in common a fully differentiable and layer-structured architecture. Depending on the number of hidden layers, the networks can be considered as shallow or as deep. From a mathematical point of view, one of the key ingredient that makes NN so efficient is their ability to integrate differentiable operations well suited to the structure of the data (convolution for images, recurrent units for time series, attention mechanisms for sequences etc.).

In this paper, we used some of the latest tools from deep learning, such as residual connections^38^, batch normalization^39^, and dropout^40^, into fully connected NN.

In particular, we studied separately the cases:

- Dense NN with one fully connected hidden layer, but with a variable number of neurons.
- Dense NN with different numbers of fully connected hidden layers, all composed by 64 neurons.
- Dense NN with different odd numbers of fully connected hidden layers, all composed by 64 neurons, with full pre-activated residual blocks^41^

All models have been implemented in Python using the library Keras^42^ running on top of TensorFlow^43^.

#### Gradient boosting on decision trees

Boosting is a meta-algorithm based on the idea of gradually aggregating numbers of simple algorithms, called weak learners, to get a final strong learner^44^. More specifically, each weak learner is optimized to minimize the error on the training data using the sum of the previous weak learners predictions as an additional input.

Based on the seminal work of Friedman^45^ who introduced gradient boosting of decision trees (in fact CART trees), several implementations have been recently developed. In this paper, we compared the three most popular ones: XGBoost^46^, LightGBM^47^ and CatBoost^48^. While built on structurally similar ideas, these libraries slightly differ on how decision trees are grown or how categorical variables data are handled, and only experimentation can validate which performs best. To implement these models, we used the corresponding Python packages.

#### Random

In order to correctly identify the properties of the different models, we built a “random” model in which random weights are given to the preselected SNPs. In particular, this model allowed us to address the question of feature importance selection by taking into account biases related to the distribution of SNPs on the Immunochip. The AUC for this model was around 0.5 for any fold of the dataset.

### SNP, loci and features importance selection

The large number of SNP markers and their not uniform distribution along the genome (see Supplementary Fig. S1), open the problem of categorizing them into functionally separated loci. Conventionally, signals from different markers are defined as coming from the same locus if the corresponding SNPs lie within a certain physical/genetic distance of each other. In order to simplify the analysis of the ordered lists of feature importance and based on the work by Jostins *et al*.^3^, we choose to define loci by globally partitioning the DNA in windows with sizes of 500 kb, which they prove to be a good trade-off between the need for functional independence of the genetic signals and the risk of splitting SNPs acting on the same gene in two independent loci. If two different SNPs lie in the same window, we consider them as belonging to the same locus.

We addressed the question of the important features selection. For each class of models we chose a paradigmatic one: Lasso penalized LR, a Residual Dense NN with 3 hidden layers of 64 neurons (ResDN3) and LightGBM (LGBM) for GBT. We then retained a criterion to assign a score of importance to each original feature (SNP).

Permutation feature importance (PFI) score is a widely used criterion: Random permutations on the samples have been performed at the level of each feature on the test set, worsening the AUC final score. The larger the deviation from the original AUC, the highest was the rank importance of the feature. For each features, the final score is obtained after averaging over the scores given by 10 different permutations. The main advantage of PFI score is that it can be universally used, independently on the ML model.

Moreover, in order to distinguish the dependence of the results on the model from that due to the criterion used to assign the ranking, we considered also a different criterion for LR and LGBM models. For LR we used the absolute value of the weight associated to each feature. The higher this value after training, the most important the corresponding feature was considered. For LGBM we used the ‘gain’ option already implemented in the library (it measures the average gain of the feature when it is used in trees).

A given model, with a fixed criterion, trained on different subsets of the data, give different results for the feature importance scores. To take into account this variability, for the second part of our analysis we created 10 different folds: starting from the original whole dataset, we arbitrarily permuted 10 times the data and re-divided the dataset in Train and Test sets (with the same proportion of samples). For each model we then considered 10 different lists of feature importance.

Since close SNPs can be considered as not independent, we compared the importance criteria of loci rather than of SNPs. To do that, we assigned a rank to the loci consisting of the highest score of the SNPs located in the genetic region. We then compared the predictions of the different models between them and with the loci identified by the meta-analysis of^3^ as associated to CD (called GWAS loci in the following). The score for these loci was assigned in the same way described above, by using the absolute value of the logarithm of OR.

Notice that in Jostins *et al*.^3^, a locus was defined as a genetic region of 500 kb around the best associated SNP. The use of the same definition of locus was not possible in our study, because we wanted to compare different lists of feature importance. Loci defined in a *relative* way to the most important SNPs of each list would not allow a direct comparison between two different lists. On the other hand, with our *absolute* definition of loci, the comparison between feature importance lists can suffer from boundary biases. In order to take into account these biases, and to smooth the comparison with the analysis of Jostins *et al*.^3^, we created a second partition (called bis) of the genome, shifted by 250 kb with respect to the original one. We then compared the two lists of SNPs coming from the two partitions (original vs original; bis vs bis and bis vs original).

Finally, the correlation between ranked lists was evaluated by a robustness measure introduced in^49^. The robustness is defined as:

1 $$R=\frac{{{\rm{\Sigma }}}_{i=1}^{x}{a}_{i}}{Mx}$$

where *M* is the number of batches of data, and if ${Q}_{i}^{x}$ denotes the first *x* features in the feature ranking *Q*_*i*_ produced by a feature selection algorithm using the *i*-th batch of data, the appearance times of each feature in the feature ranking matrix *Q*^*x*^ are counted and for the top *x* appeared features, their appearance times are ${a}_{i},i=1,2,\ldots ,x$.

We also used the Spearman rank test to evaluate the same correlation, and the results are shown in the Supplementary Information files.

Intra-model correlations were evaluated by computing the robustness *R* and Spearman rank correlation coefficients *r*_*s*_ associated to each couple of ranked lists produced by a given model on different folds. With 10 folds this gave rise to 45 *r*_*s*_ values for each model. We thus computed the mean values and standard errors. Correlation between models has been evaluated by computing *R* and *r*_*s*_ between ranked lists produced by two different models on the same fold (for a total of 10 different *R* and *r*_*s*_ values).

### Accession codes

Data have been deposited in the NCBI database of Genotypes and Phenotypes under accession numbers phs000130.v1.p1 and phs000345.v1.p1. Details about code and hyper-parameters are within the paper and its Supporting Information files. Feature importance analysis can be found at: https://github.com/romagnoni/feat_imp_GWAS.git.

## Results

We present here the results obtained by applying several ML classification methods to the IIBDGC Immunochip dataset, in terms of AUC scores and features selection after training. We first report the impact of QC and imputation strategies on the performances of the classification algorithms, in the framework of Lasso LR. We then show the results obtained by the same linear models under different regularizations. Next, we describe the results obtained by powerful and popular algorithms, GBT and NN, shallow and deep. Finally, the comparisons between the best features identified by each method are reported.

### Linear models

Different algorithms have been applied to the genotype/phenotype association problem in the literature. Besides the univariate models (based on the p-values on single SNP association tests), the simplest multivariate analyses use linear models in order to associate a phenotype to a genotype. LR is a common choice, usually coupled with Lasso regularization, after preselecting SNPs under mild constraints, as discussed in the Materials and Methods section. We compared this classical method with other linear models, in particular by changing the penalty in the regularization part of the cost function, and the preselection constraints.

#### Data pre-processing

We first studied the effect of different QC, imputation and coding strategies on AUC scores and on the selected predictor SNPs. To be able to compare the different analyses and evaluate the effects due to data pre-processing, we always applied the same algorithm, namely a LR with Lasso penalty, after SNP preselection as in the benchmark choice (${\rm{MAF}} > 0.01$ and $p < {10}^{-4}$, see Materials and Methods). The results are shown in Table 1.

**Table 1:** Impact of Quality Control, Imputation of missing genotypes and Coding methods on the results of Lasso penalized Logistic Regression.

	AUC train	AUC test	${{\boldsymbol{N}}}_{{\bf{S}}{\bf{N}}{\bf{P}}}^{{\boldsymbol{p}}}$	*N*_SNP≠0_	${{\boldsymbol{I}}}_{{\bf{S}}{\bf{N}}{\bf{P}}}^{(\ast )}$	${{\boldsymbol{I}}}_{{\bf{L}}{\bf{o}}{\bf{c}}{\bf{i}}}^{(\ast )}$	${{\boldsymbol{I}}}_{{\bf{t}}{\bf{o}}{\bf{p}}{\bf{S}}{\bf{N}}{\bf{P}}}^{(\ast )}$	${{\boldsymbol{I}}}_{{\bf{t}}{\bf{o}}{\bf{p}}{\bf{L}}{\bf{o}}{\bf{c}}{\bf{i}}}^{(\ast )}$	${{\boldsymbol{I}}}_{{\bf{L}}{\bf{o}}{\bf{c}}{\bf{i}}}^{({\bf{G}}{\bf{W}}{\bf{A}}{\bf{S}})}$	${{\boldsymbol{I}}}_{{\bf{t}}{\bf{o}}{\bf{p}}{\bf{L}}{\bf{o}}{\bf{c}}{\bf{i}}}^{({\bf{G}}{\bf{W}}{\bf{A}}{\bf{S}})}$
NoQC/Unkw/OHE	0.925 ± 0.003	0.922	23583	2927	29%	55%	6%	35%	88%	19%
QC/Unkw/OHE	0.808 ± 0.008	0.802	21896	3198	69%	87%	38%	48%	89%	25%
NoQC/Maj/sum	0.901 ± 0.003	0.897	23583	3419	36%	69%	6%	23%	90%	12%
QC/Maj/sum	0.805 ± 0.008	0.800	21896	3553	91%	100%	64%	63%	91%	27%
NoQC/HW_*c*_/sum	0.812 ± 0.007	0.803	23583	2730	38%	66%	26%	45%	87%	29%
**QC/HW**_***c***_/**sum**	**0.803 ± 0.008**	**0.800**	**21896**	**2575**	—	—	—	—	**89**%	**36**%
QC/HW_*c*_/OHE	0.796 ± 0.008	0.786	21896	3242	72%	89%	49%	60%	89%	29%
QC/HW_*c*_/raw	0.800 ± 0.008	0.792	21896	2757	72%	88%	57%	68%	89%	29%
QC/HW_*a*_/sum	0.803 ± 0.008	0.799	21896	2579	94%	99%	91%	96%	89%	36%

Area Under Curve (AUC) obtained for 10-fold cross-validation on Train set and evaluation on the Test set, for Lasso penalized Logistic Regression applied to different combinations of QC/imputation/coding choices (notations as in Materials and Methods section). The line with bold characters corresponds to our benchmark case (QC/HW_*c*_/sum). ${N}_{{\rm{SNP}}}^{p}$ indicate the number of preselected SNPs used as input of the model, ${N}_{{\rm{SNP}}\ne 0}$ is the number of SNPs associated with a nonzero coefficient. ${I}_{{\rm{SNP}}}^{(\ast )}$ and ${I}_{{\rm{Loci}}}^{(\ast )}$ refer respectively to the percentage of SNP and loci (as defined in the main test) with associated non-zero coefficient, in common with the benchmark case. ${I}_{{\rm{topSNP}}}^{(\ast )}$ and ${I}_{{\rm{topLoci}}}^{(\ast )}$ columns show the same things for the corresponding 100 features with highest weight (in absolute value). ${I}_{{\rm{Loci}}}^{({\rm{GWAS}})}$ and ${I}_{{\rm{topLoci}}}^{({\rm{GWAS}})}$ compare instead the same quantities to the list given in^3^.

The unprocessed available dataset was associated with higher AUC values suggesting that artifacts may affect the results by inflating these values. The bias was likely related to the presence of missing genotypes which may reflect remaining stratification biases (data not shown). Indeed, imputing the values of the missing alleles using the HW method resulted in a large decrease of AUC values.

SNP preselection with more stringent QC criteria limited the impact of biases. Also in this case, the HW method was the best choice to limit the impact of missing genotypes. Notice that there was no major effect of genotype imputation using the allele frequencies derived from the whole dataset or healthy controls only.

Finally, the coding method also affected the results, the higher AUC values being observed for the sum method which consists in counting the number of rare alleles contributing to the genotype.

Interestingly, the number of retained SNPs in Lasso model depended on the processing method. While a stringent QC process did not affect significantly the number of retained SNPs, the sum coding method retained less SNPs for a better result, suggesting that it extracts more information from genotypes.

Qualitatively, the retained SNPs varied a lot according to the pre-processing method. This is expected because SNPs in strong linkage disequilibrium are interchangeable if they carry a shared information. We thus looked at the loci (defined by a regions of 500 kb, see Materials and Methods section) kept by the different models. The vast majority of the predictor loci are shared by the Lasso methods when performed on the QC dataset whatever the imputation and coding strategies (Table 1).

The loci contributing to the Lasso model can be classified according to their respective weights. The best SNPs appeared often different from one model to another, whatever the data processing strategies and even for the same set of analyses. This finding argues for a large number of SNPs with comparable and low weights.

For homogeneity reasons, in the rest of the analysis we always used the QC/HWc/sum pre-processing strategy.

#### Feature and sample preselection

**Figure 2:**
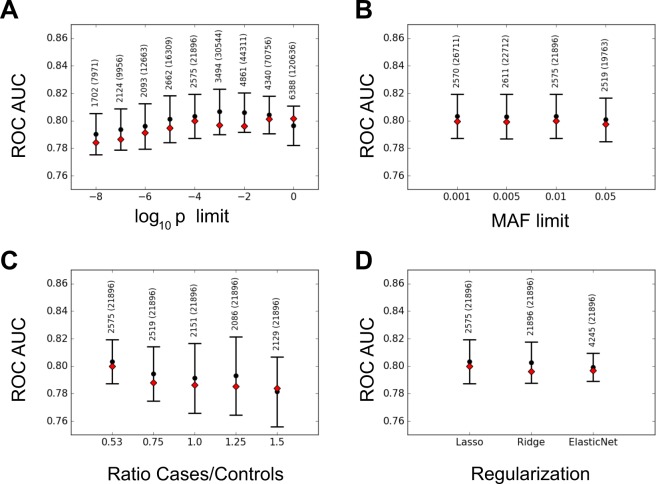
ROC AUC scores for Linear Regression model under different conditions on the dataset and on the penalty terms. Black dots and error bars refer to mean values and 2 standard deviation confidence intervals for 10 fold cross-validated models on the train dataset. Red diamonds refer to AUC scores obtained on the test dataset with the model trained on the entire train dataset, using the corresponding cross validated hyper-parameters. The numbers on top of the error bars refer to the number of features used by the model, and, in parenthesis, to the number of original features in the dataset. We show the AUC scores for: (**A**) different values of the upper bound on p-values for the SNP preselection phase, with ${\rm{MAF}} > 0.01$; (**B**) different values of the lower bound on MAF for the SNP preselection phase, with p-value $p < {10}^{-4}$; (**C**) different values of the case/control ratio; (**D**) different types of regularization. In (**C**,**D**) $p < {10}^{-4}$ and ${\rm{MAF}} > 0.01$.

To explore the impact of SNP preselection, we next performed Lasso analyses on sets of SNP which passed at least one nominal association test with a p-value threshold ranging from 10^−8^ to 1 (see Materials and Methods). The mean AUC value was not significantly affected when SNPs with p-values higher than 10^−5^ were discarded (Fig. 2A). However, even retaining only the most strongly associated SNPs (p-values lower than 10^−8^), the AUC remained higher than 0.78, despite the drastic decrease of predictors number. Indeed, the number of SNPs included in the linear model diminished from 6388 to 1702 when the threshold moved from 1 to 10^−8^. This observation suggests that the linear model is mainly built with loci having the largest nominal effect. As a consequence, when the algorithm is fed with many additional loci with very small nominal effects it does not significantly improve its ability to classify patients and controls.

We also explored the impact of preselecting SNPs on their MAF. Despite an increasing number of preselected SNPs from 19763 to 26711 when the selection threshold on MAF decreased from 0.05 to 0.001, the values of the mean AUC did not significantly changed (Fig. 2B). This result suggests that very rare alleles do not carry a large effect in the model.

In the original dataset, the case-control ratio was 0.53. To explore the impact of this unbalanced ratio on the results of Lasso LR, we changed it with alternative values ranging from 0.53 to 1.5, by eliminating controls from the dataset. The results were slightly lower, arguing for keeping all the available samples in the analyses (Fig. 2C).

Due to these results, the rest of the analyses were performed on the set of all cases and controls and for preselected SNPs with ${\rm{MAF}} > 0.01$ and association p-values lower than 10^−4^. The discarded features could in principle have a more important effect for non-linear models. Nonetheless, a similar analysis performed for the GBT algorithm LightGBM shown that no significant improvement of the AUC score is obtained when relaxing the constraints on preselection p-values (Supplementary Fig. S2). Therefore, we made the choice to keep the same preselection conditions also for all non-linear models, for consistency and to alleviate the problems related to the curse of dimensionality.

#### Regularization

To explore the impact of alternative regularization methods, we analyzed the dataset with L2 (Ridge) or mixed L1-L2 (ElasticNet) penalized regression methods (Fig. 2D). AUC values obtained with these methods were very similar to those of Lasso. However, the number of SNPs contributing to the retained models increased from 2575 (Lasso) to 4245 (Elastic net) and 21896 (Ridge). This finding suggests that the alternative methods were efficient to detect additional SNPs with very small effects but this property did not change the global result.

### Non-linear models

Non linear methods are supposed to be more efficient in detecting non linear epistatic interactions between genotypes when compared to LR methods. We explored two main categories of non linear methods based on GBT and NN.

#### Neural networks

**Figure 3:**
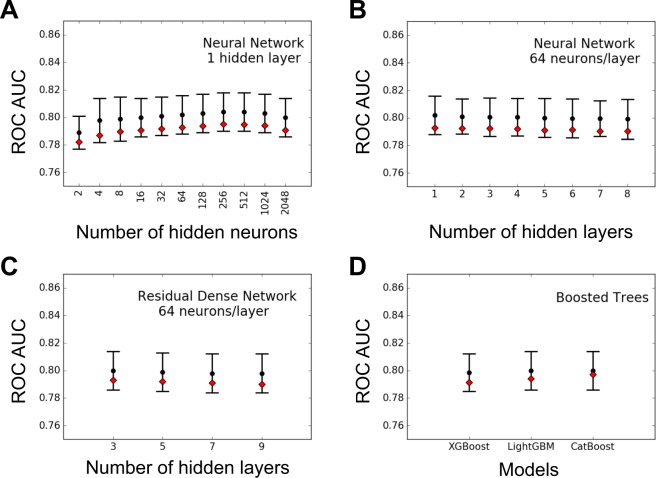
ROC AUC scores for Non-Linear models. Black dots, error bars and red diamonds are as in Fig. 2, with preselected SNP at $p < {10}^{-4}$ and ${\rm{MAF}} > 0.01$. We show the AUC scores for: (**A**) different numbers of neurons in the hidden layer of a dense NN with only one hidden layer; (**B**) different number of layers of 64 neurons, for a dense NN with multiple hidden layers; (**C**) different number of layers of 64 neurons, for a dense residual NN with pre-activation variant of residual block; (**D**) different gradient boosting for three kind of decision trees algorithms.

The simplest NNs are built with a single hidden layer composed of a variable number of neurons. The mean AUC values obtained with mono-layer NN were in the same range as LR methods (Fig. 3A). Importantly, increasing the number of hidden neurons did not significantly increase the performance in classifying patients and controls.

Inspired by the results obtained in multiple research fields by deep NN, we investigated the possibilities given by networks with multiple hidden layers (Fig. 3B). Despite the theoretically greater learning capacity of these architecture, multi-layers networks did not improve significantly the results.

It is well known though that training in deep networks can be problematic, since gradients tend to vanish in lower hidden layers. In order to take care of this problem, we implemented the idea of residual connection from^38,41^, which reduces the effect of vanishing gradients. We thus explored this more complex NN architecture, for different number of hidden layers (Fig. 3C). We obtained mean AUC values in the range of 0.80 also in this case.

#### Gradient boosting trees

GBT are a family of alternative methods proposed to go beyond linear additive models and take into account complex gene-gene interactions. We investigated three different state-of-the-art algorithms (XGBoost, LightGBM and CatBoost): As for NN, the mean AUC were in the range of 0.80 (Fig. 3D).

In summary, non linear methods did not appear more performant than linear ones arguing for limited epistatic effects in the genotyping dataset.

#### Combining the models

We investigated the possibility of combining different models in order to improve the performances. In particular we tried an ensemble method, which consists in training several classifiers and combine their predictions to check if it can outperform any single classifier. In our case, when using the average as combination rule, by combining LR, ResDN3 and LGBM we obtained ${\rm{AUC}}=0.810\pm 0.007$ on the 10-fold cross-validation, and ${\rm{AUC}}=0.802$ on the test set, thus slightly improving the results obtained with a single model. This suggests that the different models can be seen as partially complementary. Very similar results are obtained when other models considered above are included in the ensemble approach.

### Feature importance comparisons between methods

#### Comparisons with previous GWAS analyses

The IIBDGC performed a large GWAS meta-analysis which included the Immunochip dataset^3^. As a result, an association at genome-wide significance ($p < 5\cdot {10}^{-8}$) were retained for 140 independent CD loci (a locus was defined as a genetic region of 500 kb around the best associated SNP. In this paper we use a slightly different definition, see Materials and Methods) and the corresponding SNPs.

**Figure 4:**
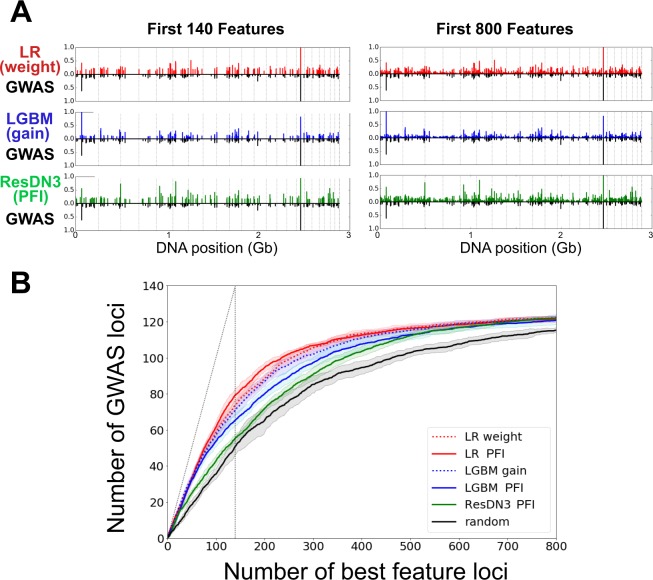
Comparison of the best features selected from different linear and non-linear models and those associated to CD in the GWAS meta-analysis by Jostins *et al*.^3^ Panel A shows the importance and the position on the genome of the best 140 (left) and 800 (right) SNPs, selected by logistic regression with Lasso regularization and weight criterion (LR weight), LightGBM with gain criterion (LGBM gain), a dense residual neural network with 3 hidden layers with permutation feature importance criterion (ResDN3 PFI), and of those reported by Jostins *et al*.(GWAS). The importance of the SNPs is given by the criteria discussed in the main text, while for GWAS we show the $|\mathrm{log}({\rm{OR}})|$. Dotted vertical lines indicate the separation between chromosomes. Panel B shows the number of common loci (as defined in the main text) between the different models with different criteria for feature selection and GWAS analysis, as a function of the first *x* selected best loci. The random model was built using randomly weighted SNPs. Solid and dotted lines represent the mean values over all the subsets, while shaded regions represent the 1 standard deviation confidence intervals. The vertical dotted line indicates the 140 limit for GWAS, while the diagonal shows the perfect agreement baseline.

We compared the set of feature SNPs identified by Jostins *et al*. and those of three methods representative of the LR, NN and GBT approaches, respectively Lasso with weight as feature importance score, LGBM with gain and ResDN3 with PFI, as described in Materials and Methods. As shown in Fig. 4A, most of the SNPs with genomic nominal significance contributed to the architecture of the ML models: SNPs with the largest OR in the analysis of^3^ also correspond to a peak for LR, LGBM and ResDN3 methods. New regions seem to consistently contribute to the different models when more features are taken into account.

We further explored if the same common pattern can be recovered at the level of loci. Figure 4B indicates that near all the first best features of the LR, LGBM and ResDN3 were among the previously reported CD-associated loci as indicated by the value close to 1, near the origin, for the slope of the “intersection” curves. Notice that this was also partially the case for the random model, were SNPs were chosen at random among the preselected SNPs. This naively unexpected large number of loci in common with the other models may be explained by observing that the density of SNPs was much higher in CD-associated regions due to the strategy of SNP selection for the Immunochip (Supplementary Fig. S1).

Far from the origin though, the first more important SNPs for all models and more importantly for non-linear models like ResDN3 and LGBM, deviate from those by GWAS studies. Nonetheless, almost all CD-associated loci reported in the analysis of  ^3^ appear in the best 800 loci for ML models, independently on the model and ranking criterion.

#### Robustness of the ML results

**Figure 5:**
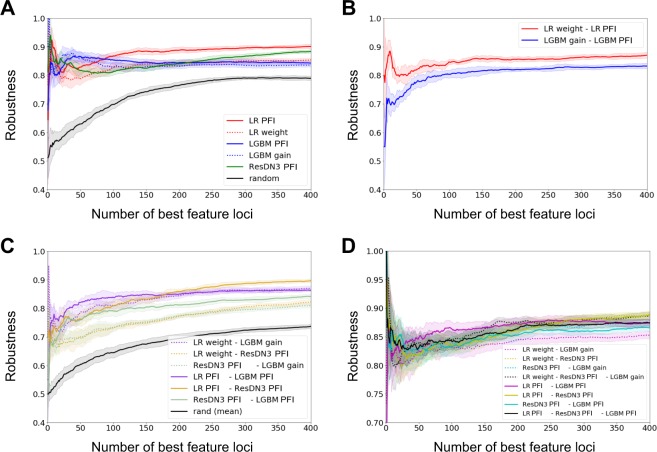
Internal and between-models coherence in feature importance selection. We show the robustness *R* as a function of the first *x* best loci. In panel (A) we consider the robustness of a given model/criterion, when trained on two different subsets of the data. In panel (B) we show the robustness between the same model when two different criteria are considered on the same subset of the dataset. In panel (C) we compare two different models/criteria, on the same subset of the dataset. Finally in panel (D) we show the same analysis of panel (A) for combination of models. Solid and dotted lines represent the mean values of the robustness distributions, respectively in panels (A and D) over all the couples of subsets (10 subsets, for a total of 45 couples), and in panels (B,C) over all the subsets (10 subsets, for a total of 10 couples). Shaded regions represent the 1 standard deviation confidence intervals.

We next questioned the robustness of the ML methods in determining the important features for this case/control classification problem. Therefore we looked at the robustness *R* of important loci arising by iterating the analyses using the same model trained on different folds of the data (Fig. 5A). A large proportion of the first tens of loci were common to all the tests. However, the proportion of loci consistently selected as important, was higher for LGBM than for LR and ResDN3 between the first ~25 and ~100 loci. Nonetheless the opposite was true starting from ~250 loci. Very similar results were obtained when considering the Spearman coefficient *r*_*s*_ (see Fig. S3 and discussion below). As expected, this proportion was significantly lower when SNPs were chosen at random.

As shown in the same Figure, the robustness through different batches of data was very similar for LGBM models with the two different criteria for feature selection (PFI and weight), while for LR the robustness for the two criteria differed in a significant way once one considered more than ~75 first best loci. Nonetheless, when compared batch by batch, weight and PFI for the LR model were more robust than gain and PFI for the LGBM model for all *x*, *x* being the number of first best loci (Fig. 5B).

The comparison between models was also performed (Fig. 5C), when trained on the same fold of the dataset. As controls, we computed also the robustness between every model and the random choice of markers, and shown the mean results over the different results. Among between-methods comparisons, LR with PFI gives the more consistent ranking with LGBM with PFI for $x\lesssim 175$ and with ResDN3 with PFI for $x\gtrsim 175$. On the other hand, ResDN3 provided features significantly more different from those of LR with weight and LGBM with gain. This observation suggests that either the measure used to weight the different loci in the NN models was not suitable, or the ResDN3 method was complementary to LR and/or LGBM.

#### Joint results

Because LR with weight, LGBM with gain and ResDN3 with PFI, could be seen as complementary, we looked at their joint results in terms of feature importance selection.

First, we looked at the loci simultaneously present within the first 140 most important of the three ML models, but absent in the GWAS meta-analysis. Two new loci were identified. rs35320439, located on chromosome 2 was in the vicinity of a gene coding for galactose-3-O-sulfotransferase 2 (*GAL3ST2*) which is over-expressed under TNF*α* stimulation in goblet cell-like *in vitro*^50^. rs395157 is located on chromosome 5, in the vicinity of a gene coding for Oncostatin M receptor (*OSMR*). Recently, high pretreatment expression of Oncostatin M was associated with failure of anti-TNF therapy in IBD patients^51^. However, retrospectively, rs35320439 and rs395157 were found nominally associated with CD ($p < {10}^{-10}$) in the studied dataset with OR values of respectively 1.158 and 1.141.

We then tested the robustness of a method able to integrate the results of two different models. We defined a list of importance features for combined methods by giving a rank to the loci in the following way. For each selected combination of models/feature selection criteria, we assigned a rank to a given locus accordingly to its order of appearance in the intersection between the ranked list of all considered models. We then evaluated their consistency throughout the training on different folds of the dataset. The mean values and standard errors are shown in Fig. 5D. Similarly to the results shown in Fig. 5A, when considered in combination with LR with PFI, the contribution of LGBM with PFI improved the consistency for ranking loci list of length between ~25 and ~150, while that of ResDN3 for $x\gtrsim 150$.

## Discussion

This work was devoted to compare the efficiency and the robustness of several ML methods.

The first part was devoted to the inspection of several technical aspects of data pre-processing which can affect the results of ML algorithms. As for statistical methods, QC constraints, imputing methods for missing genotypes and coding strategies for data analyses may affect the results of ML.

For LR methods, the very high AUC values obtained with the less stringent QC argue for a rigorous management of the raw datasets. Nonetheless, imputing the missing genotypes is also a key factor and in our analysis random imputation according to HWE corresponds to the most conservative case. Differences in these criteria may explain the discrepancies between this study and the previous report by Wei *et al*. who found a maximum AUC of 0.86 with almost the same dataset but putatively with a less conservative constraints^15^. Similarly, using methods based on random forest algorithm, Botta *et al*. also analyzed the impact of QC on the results^20^. They obtained an AUC of 0.95 with weak QC pre-processing but only 0.76 with a more stringent pre-processing. Even if their impact appears less pronounced, coding strategies may also affect the results of ML algorithms, the summation of the number of minor alleles being the most efficient in our analysis.

As an added proof for these results, we initially used a different dataset, with less stringent choices for the QC and imputation strategy. The AUC for Lasso LR was artificially inflated to 0.85 but interestingly, GBT methods outperformed these results, by 2% on this dataset. In regards to the results obtained later with more rigorous QC choices, we made the hypothesis that GBT better exploit the biased information hidden in missing values. In conclusion and not surprisingly, this analysis confirms that QC, imputing and coding methods have to be taken into account when comparing the results obtained by different groups and different methods.

For this study, we used the Immunochip dataset built through the international consortium for IBD genetics. Our choice was motivated by the fact that this dataset contains three fold more cases than the largest GWAS dataset available for CD. However, the Immunochip panel of markers is a less homogeneous representation of the genome than a GWAS panel. Chen *et al*. estimated that about 25% of the SNP heritability that is tagged in the GWAS data is lost using the ImmunoChip^52^. It can thus be imagined that some key SNPs, somewhere in the genome, common or rare, may have not been tested. However, the Immunochip panel has been built to include all the common genetic variants with nominal p-values $p < {10}^{-4}$ in previous GWAS analyses. Thus, it is supposed to contain the majority of common CD-associated SNPs. Considering that there is no example of a genetic variant with no nominal associations and with a strong effect discovered by ML algorithms, it seems unlikely that a larger panel would increase a lot the maximum AUC values. The results obtained with variable preselection thresholds on p-values and MAF also argue against putative common or rare alleles unavailable in the dataset but playing a key role in the genetic architecture of CD. Nonetheless the co-occurrence of numerous small epistatic effects cannot be excluded *a priori* and an in-depth analysis on GWAS-like datasets will be compelling in the future to confirm or exclude this possibility.

Even if we explored a large panel of ML methods, a limitation of the study is that it is based on a finite number of algorithms. Indeed, by definition, not all possible algorithms can be tested by a group alone and one could always wonder if an alternative approach could be more efficient. To tackle this question, we explored the *wisdom of the crowd* idea. 73 graduate students worked independently on the partial and biased dataset mentioned above, where names and position of SNPs and disease trait were hidden to obfuscate the data. Top performers used GBT methods, obtaining our same score values. Overall, this exercise can be seen as an external confirmation of the robustness of the results presented in this paper.

Consistently with GWAS analyses, the different ML methods recognized the CD-associated loci with the best nominal p-values among their best predictors. Although we identified two new CD-associated genes with ML methods, it appears that retrospectively, they could have been detected by classic statistical approaches. This observation thus argues against the presence of loci with no nominal effect but major effect in the classification problem. In addition to the known CD-associated loci, ML models take into account many additional SNPs with low effect. These numerous genetic variants likely carry similar impact on the classification performance as shown by their interchangeability within and between models. As a whole, these findings do not argue for large epistatic effects in the genetic architecture of the disease.

While deep learning methods have proved to be extremely efficient on unstructured signal data (images, sound, text, time-series etc.), boosting methods using decision trees as weak learners remain the non-linear default algorithm used by ML practitioners for any other type of data. On our data the different ML methods have similar power to classify patients and controls. Also, when comparing the robustness for the lists of the most important loci for the different models, when trained and tested on different batches of the data, no model proved itself consistently better than the others. For lists of 25–75 best loci, GBM methods appear to be more robust, while LR and NN should be chosen for longer lists. As discussed in a large part of the literature, the choice of the feature importance criterium play an important role and permutation feature importance seem to be more suitable for the problem we investigated.

Under stringent QC constraints, the maximum AUC values obtained by LR, GBT or NN are in the range of 0.80. For comparison, AUC of 0.75 and 0.99 have been proposed as relevant thresholds for diagnostic classifiers clinically useful when applied respectively to at-risk people or to the general population^2^. More importantly, these modest AUC values contrast with the expected ones. Theoretical works have shown that for a complex genetic disorder with a strong heritability and a low prevalence like CD, a genomic profile would be able to reach an AUC close to 1^2^. Even if the variants included in the genomic profile explain only 1/4 of the known genetic variance, the AUC is expected to be as high as 0.86^2^. The incapacity of ML methods to reach this value could indicate that the ability to classify diseased people may not be accessible with the genetic information alone. On the other hand, in our study, the specific design of the Immunochip could have withdrawn from the very beginning SNPs responsible for nonlinear epistatic effects. In order to have a more accurate evaluation of the quantity of information about the disease which is contained in the genome, similar studies should also be performed on larger dataset, like GWAS, if provided with a sufficient cohort. Nonetheless, the incorporation of environmental parameters and/or phenotypic information like RNA levels or protein functions in ML methods could also be necessary to reach the goal of disease prediction.

## Supplementary information

supplementary information
