# Supplementary material for: Comparative performances of machine learning methods for classifying Crohn Disease patients using genome-wide genotyping data
Source: Sci Rep. 2019 Jul 17;9:10351. doi: 10.1038/s41598-019-46649-z (PMC6637191; doi:10.1038/s41598-019-46649-z)
Supplement: Supplementary file 1 — supplementary information [file 41598_2019_46649_MOESM1_ESM.pdf]

Comparative performances of machine learning methods for classifying Crohn Disease patients using genome-wide genotyping data.

Supplementary Information Text

Alberto Romagnoni, Simon Jégou, Kristel Van Steen, Gilles Wainrib, Jean-Pierre Hugot
and International Inflammatory Bowel Disease Genetics Consortium (IIBDGC)

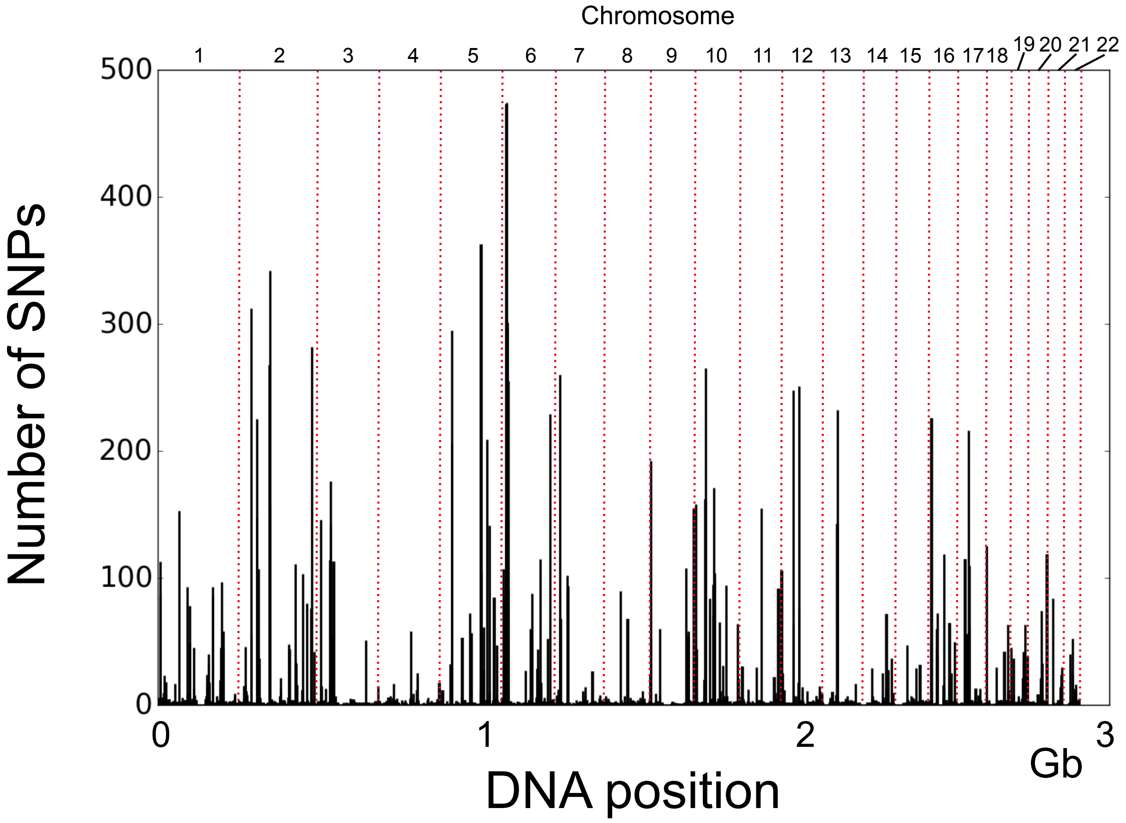

Fig. S1. Preselected SNPs density on DNA.
We show the number of SNPs per window of 500 kb, as a function of the position in the DNA (units are Gb = 10^9 bases pairs). Dotted red vertical lines indicate the separation between different chromosomes. Chromosome labels are also indicated on the top of the figure.

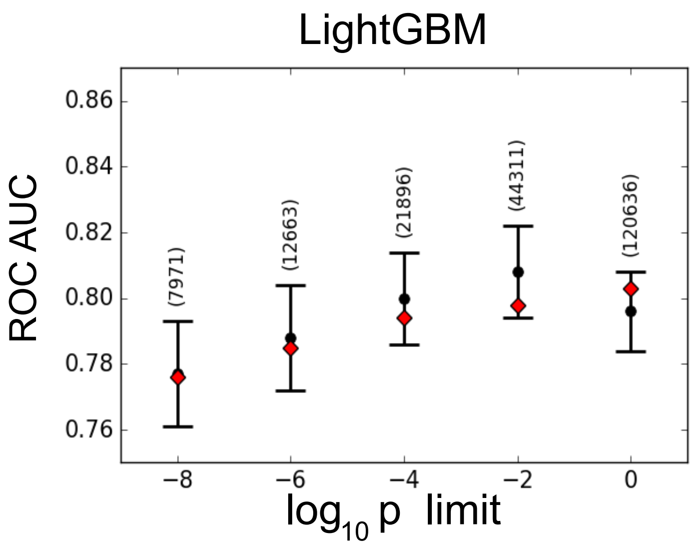

Fig. S2. ROC AUC scores for LightGBM models, for different preselections.
Black dots and error bars refer to mean values and 2 standard deviation confidence intervals for 10 fold cross-validated models on the train dataset. Red diamonds refer to AUC scores obtained on the test dataset with the model trained on the entire train dataset, using the corresponding cross validated hyper-parameters. The numbers in parenthesis on top of the error bars refer to the number of original features in the dataset. We show the AUC scores for different values of the upper bound on p-values for the SNP preselection phase.

Details about hyper-parameters optimization, settings and architectures

The hyper-parameters of the different models have been optimized through standard 10-fold cross-validation (CV) on the Train test. To have control on overfitting, each model has been trained for different sets of parameters, and optimal values have been defined as those that maximize the mean AUC values over the validation folds. These optimal values have been then kept for the final models trained on the whole Train set and tested on the Test set.

Logistic Regression

We have used Scikit-Learn [1] classifier *sklearn.linear_model.LogisticRegression* and fixed by CV the hyper-parameters:

- **C_1**: inverse of regularization strength for l1 penalization,
- **C_2**: inverse of regularization strength for l2 penalization.

For the ElasticNet case, we used *sklearn.linear_model.SGDClassifier* and CV over the hyper-parameters:

- **alpha**: a constant that multiplies the regularization term,
- **l1_ratio**: the Elastic Net mixing parameter.

We list in the following the hyper-parameter values which gave the best mean AUC score under CV.

Pre-processing -- Table 1

NoQC / Unkw /OHE: **C_1**= exp(-3.5)
NoQC / Maj /sum : **C_1**= exp(-3)
NoQC / HW_c /sum : **C_1**= exp(-3.5)

QC/ Unkw /OHE : $\mathbf{C_1}$= exp(-3.5)
QC/ Maj /sum : $\mathbf{C_1}$= exp(-3)
QC/ HW_a /sum : $\mathbf{C_1}$= exp(-3.5)
QC/ HW_c /raw : $\mathbf{C_1}$= exp(-3.5)
QC/ HW_c /OHE : $\mathbf{C_1}$= exp(-3.5)
QC/ HW_c /sum : $\mathbf{C_1}$= exp(-3.5)

Different pre-selection p-values thresholds (MAF>0.01) -- Fig. 2A

Pre-selection $p<10^{-8}$: $\mathbf{C_1}$= exp(-2.5)
Pre-selection $p<10^{-7}$: $\mathbf{C_1}$= exp(-2.5)
Pre-selection $p<10^{-6}$: $\mathbf{C_1}$= exp(-3)
Pre-selection $p<10^{-5}$: $\mathbf{C_1}$= exp(-3)
Pre-selection $p<10^{-4}$: $\mathbf{C_1}$= exp(-3.5)
Pre-selection $p<10^{-3}$: $\mathbf{C_1}$= exp(-3.5)
Pre-selection $p<10^{-2}$: $\mathbf{C_1}$= exp(-3.5)
Pre-selection $p<10^{-1}$: $\mathbf{C_1}$= exp(-4)
Pre-selection $p<10^{-0}$: $\mathbf{C_1}$= exp(-4)

Different pre-selection MAF thresholds ($p<10^{-4}$) -- Fig. 2B

Pre-selection MAF > 0.001 : $\mathbf{C_1}$= exp(-3.5)
Pre-selection MAF > 0.005 : $\mathbf{C_1}$= exp(-3.5)
Pre-selection MAF > 0.01 : $\mathbf{C_1}$= exp(-3.5)
Pre-selection MAF > 0.05 : $\mathbf{C_1}$= exp(-3.5)

Different ratios Cases/Controls -- Fig. 2C

ratio cases/controls = 0.53 : $\mathbf{C_1}$= exp(-3.5)
ratio cases/controls = 0.75 : $\mathbf{C_1}$= exp(-3.5)
ratio cases/controls = 1 : $\mathbf{C_1}$= exp(-3.5)
ratio cases/controls = 1.25 : $\mathbf{C_1}$= exp(-3.5)
ratio cases/controls = 1.5 : $\mathbf{C_1}$= exp(-3.5)

Different regularization -- Fig. 2D

Lasso (l1) : $\mathbf{C_1}$ = exp(-3.5)
Ridge (l2) : $\mathbf{C_2}$ = exp(-7.5)
ElasticNet (l1+l2) : **alpha** = exp(-3.15), **l1_ratio=** exp(-4)

Neural Networks

Neural networks have been implemented in Keras [2] on top of Tensorflow [3]. In particular we made use of Keras functional API and of pre-implemented layers Input, Dense, Activation, BatchNormalization, Dropout and Add.
We used Adam optimizer, with learning rate lr and default parameters choice beta_1=0.9, beta_2=0.999, epsilon=1e-08 and loss='binary_crossentropy'. We used 312 as batch size for training.
Although for all final models we used a fixed value for lr, we also tried learning rate schedules (based on epochs, or based on performance on the validation set), but this did not improve the results.

Dense NN with one fully connected hidden layer, but with a variable number of neurons

We used the architecture:

Input
Dense(N_h, kernel_initializer='glorot_uniform'),
BatchNormalization
Activation('sigmoid')
*Dropout(**d**)*
*Dense(1, activation='sigmoid', kernel_initializer='glorot_uniform', kernel_regularizer=l1(**C**))*

We optimized under CV the learning rate **lr**, the dropout coeffient **d**, the l1 regularization parameter **C**, and the number **E** of epochs for training. We obtained **lr**= 0.00001**, d** = 0.3 and:

For N_h=2 : **C** = 0.01 , **E** = 100
For N_h=4 : **C** = 0.01 , **E** = 75
For N_h=8 : **C** = 0.01 , **E** = 75
For N_h=16 : **C** = 0.001, **E** = 75
For N_h=32 : **C** = 0.1 , **E** = 75
For N_h=64 : **C** = 0.01 , **E** = 50
For N_h=128 : **C** = 0.1 , **E** = 75
For N_h=256 : **C** = 0.1 , **E** = 100
For N_h=512 : **C** = 0.1 , **E** = 100
For N_h=1024 : **C** = 0.1 , **E** = 25
For N_h=2048 : **C** = 0.001, **E** = 25

We also checked that already for N_h=2 the model could overfit training data after few thousand epochs.

Dense NN with different numbers of fully connected hidden layers, all composed by 64 neurons

We used the architecture:

Input
for i in range(N_L):
  Dense(64, kernel_initializer='glorot_uniform'),
  BatchNormalization
  Activation('sigmoid')
  *Dropout(**d**)*
*Dense(1, activation='sigmoid', kernel_initializer='glorot_uniform', kernel_regularizer=l1(**C**))*

We optimized under CV the number **E** of epochs for training. We fixed **lr**= 0.00001**, d** = 0.3, **C** = 0.1 and

For N_L =2 : **E** = 50
For N_L =3 : **E** = 50
For N_L =4 : **E** = 50
For N_L =5 : **E** = 50
For N_L =6 : **E** = 50
For N_L =7 : **E** = 75
For N_L =8 : **E** = 75

Dense NN with different odd numbers of fully connected hidden layers, all composed by 64 neurons, with full pre-activated residual blocks

By defining a block h_i = B (H_{i-1}) as the sequence:

for j in range(2):
  BatchNormalization
  Activation('sigmoid')
  *Dropout(**d**)*
  Dense(64, kernel_initializer='glorot_uniform'

with input H_{i-1} and output $h_{i,}$ we used the architecture:

Input
H_0 = Dense(64, kernel_initializer='glorot_uniform'),
for i in range(1,N_B):
  h_i= B(H_{i-1})
  H_i=Add(H_{i-1}, h_i)
*Dense(1, activation='sigmoid', kernel_initializer='glorot_uniform', kernel_regularizer=l1(**C**))*

We optimized under CV the number **E** of epochs for training. We fixed **lr**= 0.00001**, d** = 0.3, **C** = 0.1 and

For N_B = 1 (3 hidden layers) : **E** = 125
For N_B = 2 (5 hidden layers): **E** = 100
For N_B = 3 (7 hidden layers): **E** = 75
For N_B = 4 (9 hidden layers): **E** = 75

Gradient Boosting Trees

We used classifiers of XGBoost [4] ('objective':'binary:logistic'), LightGBM [5] ('objective': 'binary', 'metric':'binary_logloss') and CatBoost [6] (CatBoostClassifier, loss_function='Logloss').
All these models have tenth of parameters to tune, but we focused CV only on those which are known to affect the most the final score. We left the others at their default values.
We list in the following the hyper-parameter values which gave the best mean AUC score under CV.

XGBoost -- Fig. 3D

num_round=10000,
eta=0.01,
min_child_weight = 8,
alpha=0.5,
lambda= 5,
gamma = 1,
subsample=0.7,
colsample_bytree = 0.9.

LightGBM -- Fig. 3D

num_round=10000,
learning_rate =0.05,
max_depth= 8,
lambda_l1= 0.1,
lambda_l2 =1,
num_leaves = 25,
feature_fraction= 0.6.

Catboost -- Fig. 3D

iterations=10000,
learning_rate=0.05,
depth=4,
l2_leaf_reg=5.

Complementary results on feature importance selection

In the main text, we have studied the importance of the features selected by the different models when the same criterion (permutation feature importance score) is used or when other ranking scores (weights or gain) are considered. In this section, we show some complementary results concerning the problem of determining the most important features for the Crohn case-control study.

Dependence on the number of permutations

The permutation feature importance score is obtained by randomly permuting on the at the level of each feature on the test set. The larger the deviation from the original AUC, the highest was the rank importance of the feature. However, this result is not very reliable at the level of a single permutation, because random effects related to the limited sample size can affect the score. Therefore, the final score for each feature appear to be more consistent when obtained after averaging over the scores given by several different permutations. In Fig. S2 we show the results for the Spearman rank test on logistic regression with Lasso regularization, LightGBM and the ResDN3 neural network, respectively for the first 50 and 400 loci, when the number of permutations per features over which we averaged is varied. One can see how around N=10 permutations the results seem to converge to stable values.

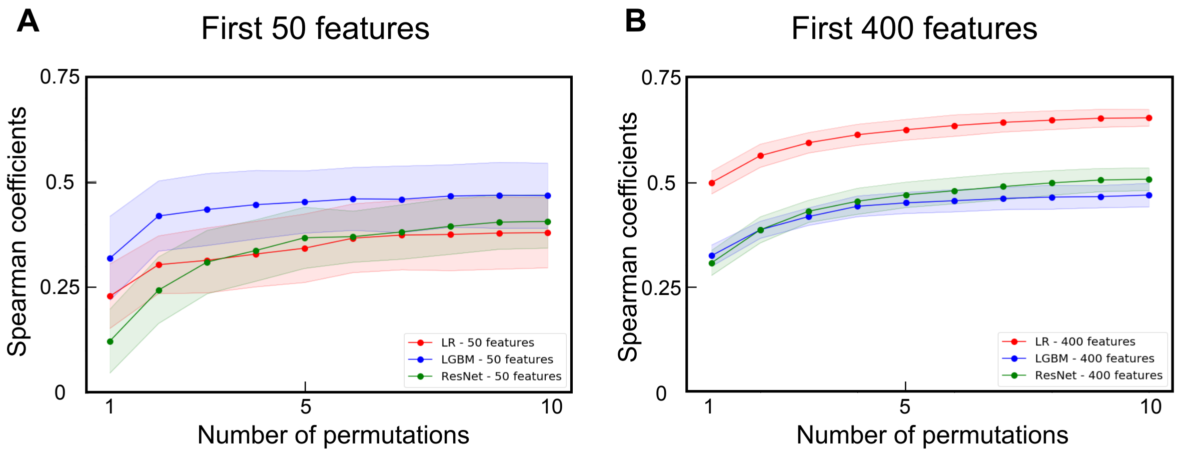

Fig. S2. Permutation feature importance scores.
We show the Spearman Rank test coefficient r_s respectively for the best 50 (panel A) and 400 (panel B) loci, as a function of the number N of permutations per feature. The features ranks are given after averaging over the N permutation feature importance scores. We show the results for logistic regression with Lasso regularization (LR), LightGBM (LGBM), and a dense residual neural network with 3 hidden layers (ResDN3). We consider the intersection between the same kind of models, when trained on two different subsets of the data. Solid lines represent the mean values, respectively over all the couples of subsets (10 subsets, for a total of 45 couples), while shaded regions represent the 1 standard deviation confidence intervals.

Spearman rank Test

In the main text, we compared the stability of the feature importance scores for the different models under study, in terms of the robustness *R*. In Fig. S3 we show the same results in term of the Spearman rank test r_s coefficients, as a function of the first *x* best loci.
In Fig. S3A, for a given model, we computed r_s for the ranks given by the same criterion applied to two different subsets of training data. To compare the results for two different criteria when the same model is trained on a given subset of data, in Fig. S3B the r_s coefficients when comparing LR with PFI and weight, and LGBM with PFI and gain are shown.

In Fig. S3C we studied the consistency of the rankings given by two models (with given criterion) on the same subset of training data. Finally, in Fig. S3D we evaluated the consistency of combined models on couples of subsets of training data, as defined in the main text. All in all, the results shown are qualitatively the same of those of Fig. 5 in the main text, but on a different scale, indicating the interchangeability of robustness *R* and Spearman r_s coefficient as measures to estimate the consistency of the feature importance ranking.

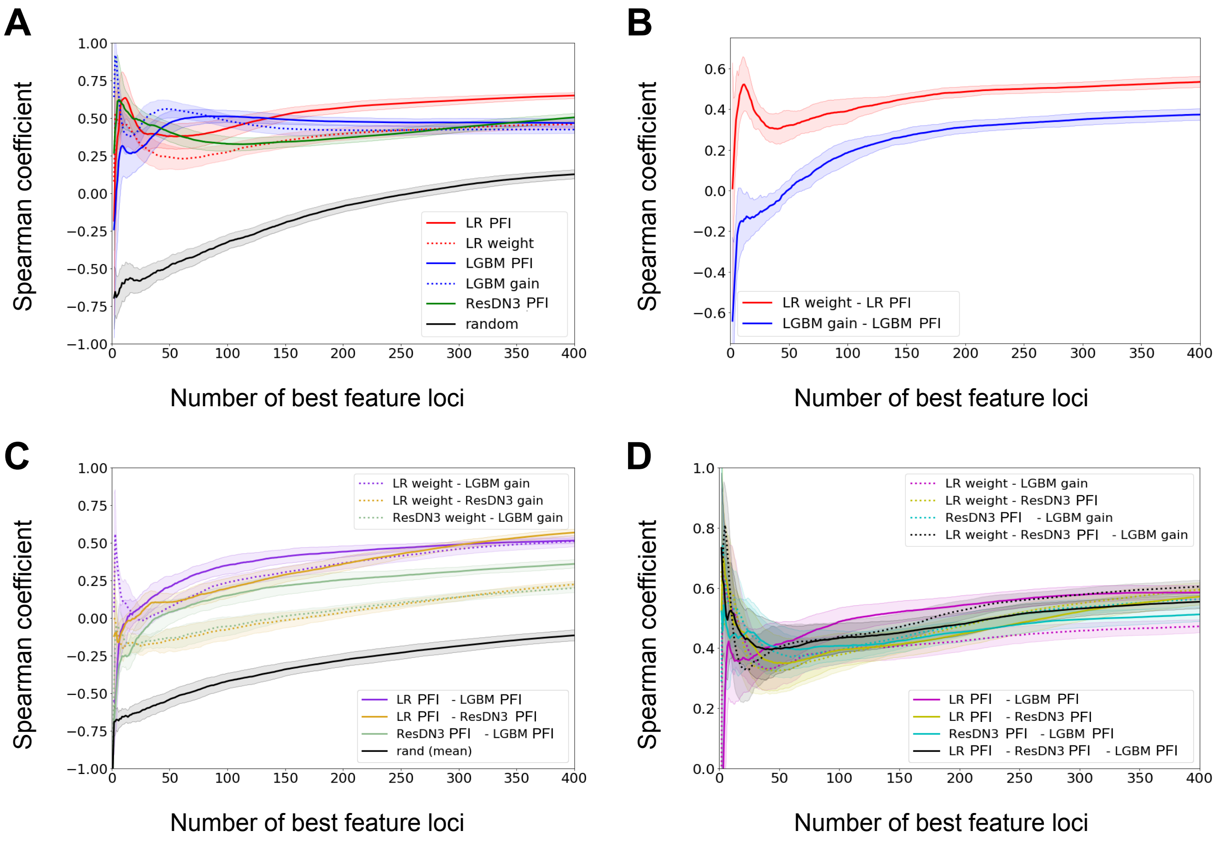

Fig. S3. Comparison between permutation feature importance and other ranking scores.
We show the Spearman Rank test coefficient r_s as a function of the first x best loci. In panel A we consider the robustness of a given model/criterion, when trained on two different subsets of the data. In panel B we show the robustness between the same model when two different criteria are considered on the same subset of the dataset. In panel C we compare two different models/criteria, on the same subset of the dataset. Finally in panel D we show the same analysis of panel A for combination of models.
Solid and dotted lines represent the mean values of the robustness distributions, respectively in panel A and D over all the couples of subsets (10 subsets, for a total of 45 couples), and in panel B and C over all the subsets (10 subsets, for a total of 10 couples). Shaded regions represent the 1 standard deviation confidence intervals.

We resumed the results given by the Spearman Rank Test for the 400 best loci in the heat-maps of Fig. S4. In particular, we separated the results given by different models but the same feature selection criterion (Fig. S4A), to mixed scenarios (Fig. S4B). Once again the results were very similar to those obtained in terms of robustness *R* in Fig. 3A.

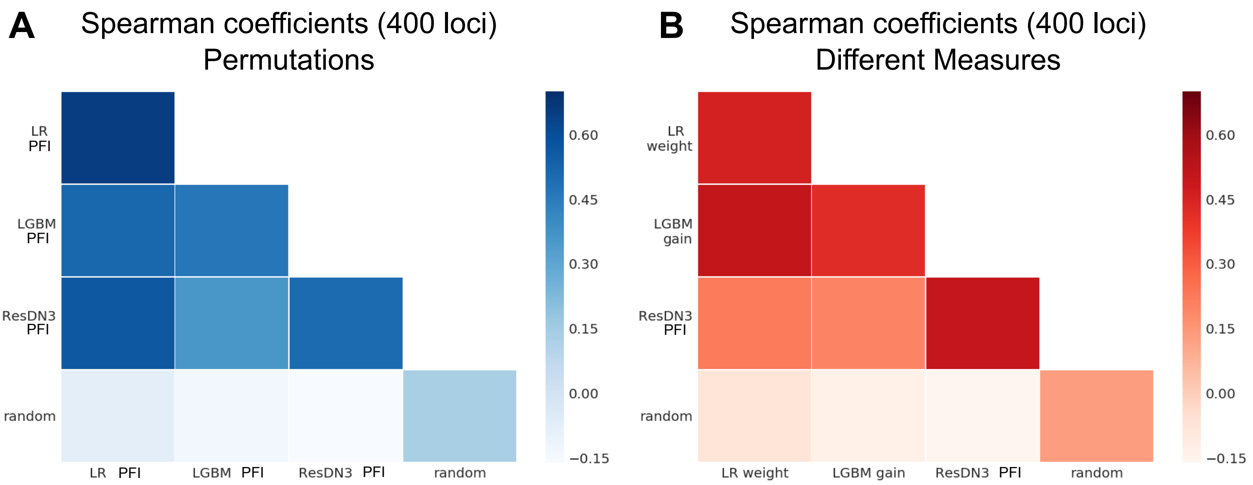

Fig. S4. Spearman rank coefficient for lists of the 400 most important loci.
We show the Spearman rank correlation coefficients for within- and between- models couples of lists of the 400 most important loci. On the diagonal are the results for the same model trained on two different subsets of the data (45 different couples of lists). Off-diagonal, are the results for two lists generated by two different models trained on the same subset of data (10 different couples of lists). Only mean results are shown. In the heat-map of panel A the same criterion (PFI) is used for all models, while in panel B we considered mixed scenarios.

Order	LR weight	LR PFI	LGBM gain	LGBM PFI	ResDN3 PFI
1	chr16, 50750kb	chr16, 50750kb	chr16, 50750kb	chr7, 26750kb	chr16, 50250kb
2	chr5, 40250kb	chr5, 40250kb	chr1, 67750kb	chr6, 31750kb	chr16, 50750kb
3	chr1, 67750kb	chr7, 26750kb	chr16, 50250kb	chr6, 32750kb	chr6, 32250kb
4	chr7, 26750kb	chr1, 67750kb	chr2, 27750kb	chr1, 67750kb	chr2, 242750kb
5	chr6, 31750kb	chr6, 32250kb	chr10, 101250kb	chr16, 50750kb	chr5, 38750kb
6	chr16, 50250kb	chr10, 101250kb	chr10, 64250kb	chr12, 57750kb	chr20, 57750kb
7	chr6, 32250kb	chr5, 158750kb	chr2, 25250kb	chr16, 50250kb	chr1, 67750kb
8	chr10, 101250kb	chr16, 50250kb	chr5, 40250kb	chr5, 40250kb	chr10, 101250kb
9	chr5, 150250kb	chr6, 167250kb	chr6, 31750kb	chr8, 126750kb	chr17, 25750kb
10	chr5, 158750kb	chr10, 35250kb	chr12, 40750kb	chr10, 101250kb	chr8, 129750kb
11	chr22, 39250kb	chr9, 117250kb	chr10, 82250kb	chr17, 40250kb	chr21, 34750kb
12	chr19, 10250kb	chr17, 37750kb	chr5, 38750kb	chr5, 38750kb	chr6, 167250kb
13	chr6, 167250kb	chr22, 39250kb	chr3, 49750kb	chr3, 18750kb	chr14, 75750kb
14	chr14, 75750kb	chr2, 242750kb	chr2, 242750kb	chr10, 35250kb	chr20, 42750kb
15	chr10, 35250kb	chr9, 4750kb	chr9, 4750kb	chr7, 51250kb	chr7, 26750kb
16	chr6, 32750kb	chr6, 32750kb	chr17, 25750kb	chr5, 158750kb	chr5, 55250kb
17	chr13, 42750kb	chr5, 38750kb	chr6, 31250kb	chr6, 167250kb	chr6, 32750kb
18	chr2, 242750kb	chr4, 123250kb	chr2, 234250kb	chr2, 242750kb	chr20, 62250kb
19	chr4, 123250kb	chr20, 62250kb	chr11, 76250kb	chr22, 39250kb	chr10, 64250kb
20	chr3, 18750kb	chr16, 28750kb	chr7, 26750kb	chr1, 160750kb	chr11, 65750kb
21	chr8, 129750kb	chr6, 31250kb	chr5, 158750kb	chr15, 38750kb	chr5, 156250kb
22	chr17, 37750kb	chr11, 76250kb	chr6, 106250kb	chr8, 129750kb	chr7, 50250kb
23	chr10, 6250kb	chr10, 64250kb	chr20, 62250kb	chr6, 30250kb	chr13, 40750kb
24	chr9, 117250kb	chr14, 75750kb	chr21, 34750kb	chr9, 139250kb	chr7, 100250kb
25	chr11, 65750kb	chr17, 25750kb	chr14, 75750kb	chr13, 42750kb	chr16, 28750kb
26	chr9, 4750kb	chr9, 139250kb	chr10, 35250kb	chr5, 55250kb	chr10, 94750kb
27	chr12, 57750kb	chr5, 131750kb	chr6, 21250kb	chr21, 34750kb	chr5, 158750kb
28	chr1, 1250kb	chr5, 150250kb	chr22, 39250kb	chr10, 35750kb	chr8, 27250kb
29	chr5, 38750kb	chr6, 31750kb	chr19, 10250kb	chr14, 75750kb	chr12, 57750kb
30	chr22, 30250kb	chr20, 57750kb	chr8, 129750kb	chr17, 25750kb	chr11, 134250kb
31	chr7, 98750kb	chr2, 65750kb	chr19, 49250kb	chr4, 123250kb	chr5, 71750kb
32	chr20, 62250kb	chr21, 34750kb	chr6, 32750kb	chr2, 103250kb	chr15, 63750kb
33	chr6, 31250kb	chr6, 106250kb	chr20, 57750kb	chr10, 6250kb	chr8, 79750kb
34	chr2, 25250kb	chr2, 25250kb	chr19, 750kb	chr20, 57750kb	chr22, 37250kb
35	chr13, 40750kb	chr8, 129750kb	chr16, 85750kb	chr10, 64250kb	chr9, 4750kb
36	chr2, 103250kb	chr13, 42750kb	chr7, 98750kb	chr17, 37750kb	chr15, 38750kb
37	chr20, 57750kb	chr19, 750kb	chr5, 55250kb	chr16, 28750kb	chr2, 25250kb
38	chr9, 139250kb	chr2, 103250kb	chr7, 51250kb	chr11, 65750kb	chr10, 3750kb
39	chr1, 197750kb	chr1, 200750kb	chr1, 78250kb	chr10, 59750kb	chr7, 98750kb
40	chr2, 65750kb	chr3, 49750kb	chr11, 65750kb	chr6, 32250kb	chr6, 30250kb
41	chr5, 55250kb	chr16, 85750kb	chr12, 6250kb	chr2, 25250kb	chr6, 106250kb
42	chr16, 28750kb	chr3, 18750kb	chr6, 149250kb	chr2, 65750kb	chr11, 61750kb
43	chr2, 61250kb	chr12, 57750kb	chr6, 167250kb	chr1, 206750kb	chr6, 31750kb
44	chr17, 25750kb	chr19, 34250kb	chr9, 139250kb	chr20, 62250kb	chr1, 1250kb

Order	LR weight	LR PFI	LGBM gain	LGBM PFI	ResDN3 PFI
**45**	chr6, 106250kb	chr5, 141750kb	chr17, 40250kb	chr9, 4750kb	chr19, 46750kb
**46**	chr1, 120250kb	chr11, 65750kb	chr17, 54750kb	chr1, 1250kb	chr1, 113250kb
**47**	chr1, 198750kb	chr5, 55250kb	chr2, 103250kb	chr7, 98750kb	chr19, 34250kb
**48**	chr14, 64750kb	chr6, 21250kb	chr10, 6250kb	chr11, 76250kb	chr2, 65750kb
**49**	chr2, 145250kb	chr5, 40750kb	chr10, 75750kb	chr7, 100250kb	chr6, 21250kb
**50**	chr17, 57750kb	chr2, 61250kb	chr1, 51250kb	chr1, 161750kb	chr4, 106250kb
**51**	chr11, 76250kb	chr7, 100250kb	chr7, 50250kb	chr5, 150250kb	chr1, 226750kb
**52**	chr1, 206750kb	chr2, 234250kb	chr10, 81250kb	chr2, 28250kb	chr4, 123250kb
**53**	chr19, 750kb	chr7, 50250kb	chr6, 32250kb	chr6, 21250kb	chr18, 77250kb
**54**	chr13, 107750kb	chr1, 206750kb	chr19, 34250kb	chr9, 117250kb	chr2, 27750kb
**55**	chr19, 34250kb	chr5, 156250kb	chr9, 117250kb	chr19, 10250kb	chr5, 40250kb
**56**	chr16, 85750kb	chr2, 27750kb	chr19, 33250kb	chr14, 88250kb	chr4, 187250kb
**57**	chr7, 100250kb	chr21, 16750kb	chr16, 11750kb	chr15, 67250kb	chr2, 28750kb
**58**	chr6, 105750kb	chr19, 49250kb	chr1, 206750kb	chr2, 231250kb	chr2, 61750kb
**59**	chr10, 82250kb	chr5, 71750kb	chr13, 42750kb	chr13, 40750kb	chr1, 51250kb
**60**	chr2, 28250kb	chr14, 88250kb	chr5, 131750kb	chr2, 234250kb	chr11, 35250kb
**61**	chr10, 64250kb	chr8, 126750kb	chr11, 64250kb	chr2, 145250kb	chr19, 54750kb
**62**	chr5, 71750kb	chr6, 30250kb	chr7, 100250kb	chr12, 6250kb	chr2, 201750kb
**63**	chr21, 34750kb	chr13, 40750kb	chr1, 92750kb	chr16, 11750kb	chr1, 186750kb
**64**	chr11, 134250kb	chr1, 1250kb	chr2, 28750kb	chr17, 57750kb	chr1, 206750kb
**65**	chr3, 25250kb	chr10, 6250kb	chr1, 160750kb	chr3, 49750kb	chr2, 37250kb
**66**	chr11, 33750kb	chr10, 60250kb	chr17, 57750kb	chr11, 134250kb	chr5, 173250kb
**67**	chr5, 173250kb	chr11, 60750kb	chr15, 67250kb	chr2, 27750kb	chr22, 39250kb
**68**	chr8, 79750kb	chr13, 44250kb	chr16, 28250kb	chr1, 186750kb	chr6, 149250kb
**69**	chr21, 16750kb	chr8, 27250kb	chr2, 160750kb	chr5, 156250kb	chr16, 11750kb
**70**	chr19, 33250kb	chr20, 42750kb	chr17, 37750kb	chr7, 50250kb	chr2, 28250kb
**71**	chr18, 12750kb	chr16, 11750kb	chr4, 123250kb	chr18, 12750kb	chr1, 161750kb
**72**	chr6, 30250kb	chr21, 45750kb	chr7, 148250kb	chr20, 42750kb	chr9, 117250kb
**73**	chr12, 6250kb	chr12, 6250kb	chr10, 94250kb	chr10, 94750kb	chr12, 6250kb
**74**	chr3, 49750kb	chr5, 173250kb	chr20, 42750kb	chr19, 750kb	chr5, 131750kb
**75**	chr2, 62750kb	chr21, 40750kb	chr2, 145250kb	chr1, 113250kb	chr6, 20750kb
**76**	chr15, 63750kb	chr17, 40250kb	chr2, 28250kb	chr11, 58250kb	chr2, 61250kb
**77**	chr19, 46750kb	chr19, 10250kb	chr13, 44250kb	chr7, 148250kb	chr6, 31250kb
**78**	chr7, 28250kb	chr2, 28250kb	chr18, 12750kb	chr8, 91250kb	chr5, 40750kb
**79**	chr1, 113250kb	chr7, 148250kb	chr3, 18750kb	chr5, 131750kb	chr4, 38750kb
**80**	chr2, 37250kb	chr6, 128250kb	chr3, 25250kb	chr5, 71750kb	chr9, 139250kb
**81**	chr8, 27250kb	chr2, 145250kb	chr1, 197750kb	chr6, 149250kb	chr1, 92750kb
**82**	chr14, 35750kb	chr15, 63750kb	chr11, 58250kb	chr19, 49250kb	chr9, 113750kb
**83**	chr7, 72750kb	chr5, 10750kb	chr1, 1250kb	chr15, 63750kb	chr2, 22250kb
**84**	chr1, 200750kb	chr2, 62750kb	chr8, 126750kb	chr16, 28250kb	chr14, 54250kb
**85**	chr7, 50250kb	chr8, 79750kb	chr10, 59750kb	chr11, 60750kb	chr2, 234250kb
**86**	chr5, 156250kb	chr16, 11250kb	chr5, 71750kb	chr5, 40750kb	chr11, 58250kb
**87**	chr11, 60750kb	chr1, 186750kb	chr13, 40750kb	chr19, 34250kb	chr19, 33750kb
**88**	chr2, 201750kb	chr6, 29750kb	chr2, 24750kb	chr5, 173250kb	chr10, 6250kb

Order	LR weight	LR PFI	LGBM gain	LGBM PFI	ResDN3 PFI
**89**	chr6, 21250kb	chr2, 28750kb	chr8, 91250kb	chr12, 40750kb	chr11, 96250kb
**90**	chr5, 40750kb	chr10, 94250kb	chr5, 40750kb	chr21, 16750kb	chr14, 96250kb
**91**	chr5, 141750kb	chr15, 38750kb	chr1, 161750kb	chr10, 3750kb	chr19, 37250kb
**92**	chr5, 10750kb	chr22, 30250kb	chr5, 10750kb	chr3, 49250kb	chr13, 44250kb
**93**	chr2, 234250kb	chr1, 172750kb	chr16, 28750kb	chr3, 25250kb	chr6, 29750kb
**94**	chr16, 28250kb	chr12, 40750kb	chr11, 60750kb	chr2, 37250kb	chr10, 94250kb
**95**	chr2, 136750kb	chr22, 37250kb	chr5, 156250kb	chr22, 41750kb	chr4, 26250kb
**96**	chr15, 38750kb	chr2, 231250kb	chr19, 46750kb	chr10, 82250kb	chr21, 16750kb
**97**	chr20, 42750kb	chr1, 198750kb	chr10, 94750kb	chr11, 61750kb	chr16, 10750kb
**98**	chr16, 11750kb	chr2, 241750kb	chr15, 38750kb	chr2, 61250kb	chr7, 51250kb
**99**	chr1, 172750kb	chr2, 201750kb	chr21, 16750kb	chr22, 30250kb	chr4, 48250kb
**100**	chr10, 94250kb	chr11, 134250kb	chr4, 106250kb	chr1, 51250kb	chr8, 91250kb
**101**	chr13, 53250kb	chr12, 111750kb	chr22, 41750kb	chr4, 106250kb	chr19, 750kb
**102**	chr12, 40750kb	chr7, 98750kb	chr1, 113250kb	chr22, 37250kb	chr17, 54750kb
**103**	chr6, 29750kb	chr15, 67250kb	chr22, 37250kb	chr6, 128250kb	chr11, 2250kb
**104**	chr1, 186750kb	chr10, 94750kb	chr5, 62250kb	chr7, 50750kb	chr8, 126750kb
**105**	chr22, 37250kb	chr1, 161250kb	chr2, 62750kb	chr2, 23750kb	chr2, 241750kb
**106**	chr6, 90750kb	chr1, 113250kb	chr11, 61750kb	chr7, 149250kb	chr1, 160750kb
**107**	chr3, 49250kb	chr18, 12750kb	chr12, 750kb	chr6, 106250kb	chr12, 58250kb
**108**	chr11, 58250kb	chr16, 28250kb	chr5, 150250kb	chr2, 136750kb	chr16, 11250kb
**109**	chr4, 38750kb	chr8, 91250kb	chr1, 114250kb	chr16, 85750kb	chr15, 86250kb
**110**	chr11, 4250kb	chr1, 51250kb	chr18, 20250kb	chr6, 31250kb	chr16, 85750kb
**111**	chr5, 131750kb	chr17, 54750kb	chr11, 134250kb	chr11, 128250kb	chr6, 137250kb
**112**	chr15, 67250kb	chr11, 4250kb	chr7, 149250kb	chr21, 45750kb	chr7, 83250kb
**113**	chr4, 3250kb	chr14, 96250kb	chr5, 173250kb	chr13, 107750kb	chr19, 49250kb
**114**	chr16, 11250kb	chr18, 77250kb	chr11, 2250kb	chr17, 54750kb	chr1, 198750kb
**115**	chr18, 77250kb	chr6, 30750kb	chr1, 186750kb	chr10, 94250kb	chr1, 185250kb
**116**	chr10, 90250kb	chr2, 37250kb	chr2, 23750kb	chr6, 105750kb	chr1, 205250kb
**117**	chr2, 219250kb	chr17, 57750kb	chr7, 6750kb	chr19, 33750kb	chr2, 182750kb
**118**	chr4, 106250kb	chr4, 106250kb	chr2, 61250kb	chr10, 102250kb	chr2, 102750kb
**119**	chr1, 92750kb	chr10, 81250kb	chr11, 69250kb	chr14, 96250kb	chr1, 78250kb
**120**	chr2, 86750kb	chr4, 38750kb	chr19, 34750kb	chr19, 33250kb	chr11, 64250kb
**121**	chr12, 111750kb	chr19, 46750kb	chr6, 137250kb	chr1, 172750kb	chr7, 27250kb
**122**	chr12, 56250kb	chr17, 32250kb	chr17, 46250kb	chr2, 28750kb	chr8, 116750kb
**123**	chr10, 60250kb	chr7, 28250kb	chr12, 110750kb	chr9, 128750kb	chr9, 5250kb
**124**	chr7, 148250kb	chr10, 59750kb	chr15, 63750kb	chr7, 72750kb	chr7, 148250kb
**125**	chr11, 61750kb	chr1, 67250kb	chr6, 90750kb	chr1, 78250kb	chr11, 60750kb
**126**	chr6, 137250kb	chr1, 120250kb	chr15, 86250kb	chr3, 169750kb	chr3, 101250kb
**127**	chr10, 35750kb	chr11, 64250kb	chr2, 65750kb	chr6, 134250kb	chr17, 40250kb
**128**	chr8, 126750kb	chr1, 197750kb	chr10, 35750kb	chr12, 68250kb	chr2, 160750kb
**129**	chr2, 28750kb	chr19, 33250kb	chr19, 46250kb	chr11, 4250kb	chr20, 44750kb
**130**	chr10, 3750kb	chr19, 37250kb	chr2, 181750kb	chr10, 30750kb	chr14, 39250kb
**131**	chr10, 94750kb	chr6, 90750kb	chr22, 21750kb	chr6, 20750kb	chr16, 30750kb
**132**	chr2, 23750kb	chr10, 82250kb	chr14, 96250kb	chr2, 86750kb	chr1, 55250kb

Order	LR weight	LR PFI	LGBM gain	LGBM PFI	ResDN3 PFI
**133**	chr17, 32250kb	chr4, 103250kb	chr19, 33750kb	chr11, 69250kb	chr1, 172750kb
**134**	chr14, 35250kb	chr3, 25250kb	chr10, 30750kb	chr11, 2250kb	chr5, 159750kb
**135**	chr14, 88250kb	chr19, 18250kb	chr1, 172750kb	chr16, 75250kb	chr10, 75750kb
**136**	chr2, 43250kb	chr14, 35750kb	chr8, 27250kb	chr5, 10750kb	chr11, 4250kb
**137**	chr19, 33750kb	chr2, 102750kb	chr2, 102750kb	chr12, 56750kb	chr17, 57750kb
**138**	chr3, 33750kb	chr10, 75750kb	chr1, 151250kb	chr3, 58750kb	chr1, 12250kb
**139**	chr11, 64250kb	chr1, 160750kb	chr19, 18250kb	chr1, 197750kb	chr1, 8250kb
**140**	chr1, 21750kb	chr1, 161750kb	chr5, 176750kb	chr6, 137250kb	chr10, 35750kb
**141**	chr10, 30750kb	chr10, 3750kb	chr16, 14250kb	chr2, 201750kb	chr19, 18250kb
**142**	chr14, 98250kb	chr6, 111750kb	chr6, 6750kb	chr14, 64750kb	chr3, 169750kb
**143**	chr19, 49250kb	chr11, 61750kb	chr7, 17250kb	chr2, 181750kb	chr14, 64750kb
**144**	chr16, 75250kb	chr6, 149250kb	chr10, 3750kb	chr16, 14250kb	chr2, 67750kb
**145**	chr12, 58250kb	chr1, 151250kb	chr2, 218750kb	chr19, 46250kb	chr6, 30750kb
**146**	chr18, 56750kb	chr11, 58250kb	chr6, 3250kb	chr15, 86250kb	chr1, 231250kb
**147**	chr9, 115750kb	chr3, 101250kb	chr6, 159250kb	chr11, 64250kb	chr3, 46250kb
**148**	chr6, 6750kb	chr2, 23750kb	chr11, 4250kb	chr6, 143750kb	chr10, 35250kb
**149**	chr6, 143750kb	chr14, 64750kb	chr12, 98250kb	chr6, 29750kb	chr3, 58750kb
**150**	chr9, 113750kb	chr19, 33750kb	chr6, 20750kb	chr1, 92750kb	chr17, 37750kb
**151**	chr11, 128250kb	chr4, 48250kb	chr11, 35250kb	chr16, 11250kb	chr21, 45750kb
**152**	chr17, 54750kb	chr7, 107250kb	chr16, 75250kb	chr2, 22250kb	chr8, 4250kb
**153**	chr7, 99750kb	chr22, 21750kb	chr3, 93750kb	chr11, 71250kb	chr13, 42750kb
**154**	chr1, 8250kb	chr6, 137250kb	chr4, 26250kb	chr6, 111750kb	chr3, 49750kb
**155**	chr6, 159250kb	chr1, 8250kb	chr6, 29750kb	chr11, 33750kb	chr10, 30750kb
**156**	chr8, 10750kb	chr6, 20750kb	chr4, 48250kb	chr18, 77250kb	chr16, 14250kb
**157**	chr6, 111750kb	chr2, 43250kb	chr13, 107750kb	chr7, 17250kb	chr11, 76750kb
**158**	chr20, 12250kb	chr9, 113750kb	chr7, 72750kb	chr2, 161750kb	chr1, 151750kb
**159**	chr3, 48750kb	chr9, 5250kb	chr8, 116750kb	chr18, 20250kb	chr16, 28250kb
**160**	chr1, 51250kb	chr4, 187250kb	chr14, 64750kb	chr4, 38750kb	chr2, 204750kb
**161**	chr6, 20750kb	chr10, 30750kb	chr16, 11250kb	chr5, 176750kb	chr10, 82250kb
**162**	chr6, 138250kb	chr11, 128250kb	chr14, 54250kb	chr12, 110750kb	chr17, 59250kb
**163**	chr6, 30750kb	chr1, 205250kb	chr18, 56750kb	chr8, 116750kb	chr19, 34750kb
**164**	chr8, 91250kb	chr11, 35250kb	chr4, 3250kb	chr19, 18250kb	chr2, 231250kb
**165**	chr4, 122750kb	chr6, 137750kb	chr2, 201750kb	chr1, 21750kb	chr1, 67250kb
**166**	chr2, 181750kb	chr1, 114250kb	chr9, 116250kb	chr1, 161250kb	chr1, 161250kb
**167**	chr14, 96250kb	chr18, 56750kb	chr2, 37250kb	chr12, 94750kb	chr11, 69250kb
**168**	chr13, 40250kb	chr5, 96250kb	chr11, 128250kb	chr8, 27250kb	chr22, 30250kb
**169**	chr2, 231250kb	chr7, 72750kb	chr1, 21750kb	chr17, 32250kb	chr7, 74250kb
**170**	chr10, 102250kb	chr12, 68250kb	chr2, 111750kb	chr2, 98250kb	chr7, 6750kb
**171**	chr19, 37250kb	chr20, 44750kb	chr11, 15750kb	chr10, 90250kb	chr2, 43750kb
**172**	chr6, 137750kb	chr10, 35750kb	chr2, 174750kb	chr19, 1250kb	chr2, 100750kb
**173**	chr2, 241750kb	chr4, 102750kb	chr1, 226750kb	chr10, 75750kb	chr18, 12750kb
**174**	chr1, 161250kb	chr19, 1250kb	chr4, 102750kb	chr18, 53250kb	chr6, 134250kb
**175**	chr1, 205250kb	chr2, 219250kb	chr14, 67750kb	chr4, 48250kb	chr15, 93250kb
**176**	chr12, 110750kb	chr10, 64750kb	chr20, 44750kb	chr11, 35250kb	chr7, 72750kb

Order	LR weight	LR PFI	LGBM gain	LGBM PFI	ResDN3 PFI
**177**	chr4, 187250kb	chr16, 10750kb	chr18, 77250kb	chr2, 203750kb	chr7, 20250kb
**178**	chr16, 14250kb	chr2, 136750kb	chr19, 1250kb	chr2, 185750kb	chr1, 21750kb
**179**	chr9, 128750kb	chr1, 151750kb	chr1, 68250kb	chr19, 37250kb	chr9, 116250kb
**180**	chr21, 40750kb	chr6, 143750kb	chr6, 111750kb	chr1, 55250kb	chr6, 111750kb
**181**	chr1, 200250kb	chr2, 97250kb	chr1, 92250kb	chr5, 159750kb	chr3, 48750kb
**182**	chr6, 153750kb	chr6, 250kb	chr14, 69250kb	chr1, 205250kb	chr15, 54750kb
**183**	chr7, 6750kb	chr18, 4750kb	chr2, 231250kb	chr2, 67750kb	chr11, 76250kb
**184**	chr1, 151750kb	chr16, 4750kb	chr1, 71250kb	chr14, 67750kb	chr17, 46250kb
**185**	chr6, 19250kb	chr12, 58250kb	chr16, 27250kb	chr2, 111750kb	chr1, 201250kb
**186**	chr13, 44250kb	chr6, 138250kb	chr16, 31250kb	chr6, 250kb	chr2, 174750kb
**187**	chr12, 111250kb	chr11, 2250kb	chr14, 32250kb	chr2, 102750kb	chr6, 143750kb
**188**	chr11, 35250kb	chr2, 22250kb	chr22, 41250kb	chr17, 46250kb	chr17, 40750kb
**189**	chr1, 161750kb	chr16, 14250kb	chr7, 83250kb	chr1, 200750kb	chr1, 242750kb
**190**	chr6, 149250kb	chr18, 53250kb	chr13, 40250kb	chr13, 44250kb	chr8, 57250kb
**191**	chr15, 41250kb	chr1, 185250kb	chr10, 90250kb	chr10, 81250kb	chr15, 67250kb
**192**	chr4, 26250kb	chr5, 176750kb	chr5, 141750kb	chr1, 226750kb	chr12, 40750kb
**193**	chr10, 6750kb	chr5, 72750kb	chr12, 56750kb	chr4, 100250kb	chr2, 97250kb
**194**	chr17, 40250kb	chr11, 96250kb	chr22, 30250kb	chr19, 34750kb	chr10, 81250kb
**195**	chr1, 12250kb	chr5, 159750kb	chr12, 68250kb	chr5, 141750kb	chr7, 56750kb
**196**	chr2, 191750kb	chr17, 46250kb	chr12, 40250kb	chr1, 151250kb	chr9, 34250kb
**197**	chr17, 32750kb	chr2, 67750kb	chr11, 33750kb	chr7, 6750kb	chr22, 41750kb
**198**	chr4, 103250kb	chr14, 98250kb	chr10, 6750kb	chr12, 750kb	chr11, 128250kb
**199**	chr22, 21750kb	chr11, 33750kb	chr2, 43750kb	chr12, 56250kb	chr18, 4750kb
**200**	chr1, 185250kb	chr2, 86750kb	chr15, 93250kb	chr7, 74250kb	chr19, 1250kb
**201**	chr8, 116750kb	chr4, 26250kb	chr17, 32750kb	chr21, 40750kb	chr14, 69250kb
**202**	chr10, 59750kb	chr15, 86250kb	chr11, 96250kb	chr8, 79750kb	chr9, 128750kb
**203**	chr7, 74250kb	chr18, 46250kb	chr4, 38750kb	chr22, 21750kb	chr1, 11750kb
**204**	chr12, 107250kb	chr6, 134250kb	chr6, 128250kb	chr6, 159250kb	chr6, 250kb
**205**	chr3, 169750kb	chr3, 48750kb	chr18, 47250kb	chr1, 67250kb	chr17, 32250kb
**206**	chr15, 86250kb	chr1, 92750kb	chr16, 4250kb	chr1, 151750kb	chr20, 47250kb
**207**	chr18, 4750kb	chr1, 226750kb	chr3, 169750kb	chr2, 186250kb	chr18, 53250kb
**208**	chr7, 149250kb	chr7, 6750kb	chr1, 120250kb	chr11, 96250kb	chr2, 145250kb
**209**	chr7, 56750kb	chr13, 40250kb	chr2, 86750kb	chr1, 198750kb	chr16, 4750kb
**210**	chr19, 18250kb	chr20, 43250kb	chr6, 30250kb	chr9, 34250kb	chr5, 171750kb
**211**	chr7, 83250kb	chr3, 58750kb	chr9, 34250kb	chr7, 28250kb	chr7, 17250kb
**212**	chr2, 97250kb	chr12, 68750kb	chr2, 163250kb	chr6, 137750kb	chr3, 159750kb
**213**	chr2, 111750kb	chr7, 17250kb	chr10, 125750kb	chr1, 231250kb	chr7, 149250kb
**214**	chr2, 19250kb	chr10, 6750kb	chr2, 191250kb	chr8, 10750kb	chr10, 50250kb
**215**	chr3, 101250kb	chr1, 12250kb	chr6, 134250kb	chr8, 129250kb	chr6, 138250kb
**216**	chr1, 78250kb	chr2, 43750kb	chr5, 95250kb	chr1, 200250kb	chr1, 66750kb
**217**	chr21, 45750kb	chr14, 54250kb	chr13, 53250kb	chr4, 103250kb	chr1, 197750kb
**218**	chr18, 20250kb	chr2, 160750kb	chr5, 39750kb	chr14, 98250kb	chr13, 113750kb
**219**	chr16, 87750kb	chr1, 21750kb	chr6, 138250kb	chr13, 53250kb	chr1, 200750kb
**220**	chr8, 118750kb	chr1, 173250kb	chr11, 71250kb	chr7, 107250kb	chr2, 111750kb

Order	LR weight	LR PFI	LGBM gain	LGBM PFI	ResDN3 PFI
**221**	chr1, 151250kb	chr8, 4250kb	chr5, 171750kb	chr6, 30750kb	chr2, 62750kb
**222**	chr10, 129250kb	chr8, 116750kb	chr10, 114750kb	chr14, 35250kb	chr19, 55250kb
**223**	chr1, 67250kb	chr8, 10750kb	chr11, 85750kb	chr16, 4750kb	chr2, 68750kb
**224**	chr2, 27750kb	chr6, 159250kb	chr15, 26750kb	chr1, 114250kb	chr2, 23750kb
**225**	chr13, 24750kb	chr2, 111750kb	chr12, 56250kb	chr4, 122750kb	chr13, 40250kb
**226**	chr11, 2250kb	chr14, 39250kb	chr7, 107250kb	chr4, 187250kb	chr9, 123750kb
**227**	chr1, 68250kb	chr6, 33250kb	chr12, 94750kb	chr2, 236750kb	chr14, 98250kb
**228**	chr22, 41750kb	chr18, 20250kb	chr3, 49250kb	chr11, 118750kb	chr2, 181750kb
**229**	chr10, 75750kb	chr7, 51250kb	chr6, 143750kb	chr6, 90750kb	chr1, 7750kb
**230**	chr5, 62250kb	chr3, 169750kb	chr1, 200750kb	chr4, 38250kb	chr10, 64750kb
**231**	chr11, 96250kb	chr22, 41750kb	chr3, 38750kb	chr10, 6750kb	chr2, 60750kb
**232**	chr3, 58750kb	chr21, 40250kb	chr19, 37250kb	chr10, 60250kb	chr19, 10250kb
**233**	chr1, 7750kb	chr19, 54750kb	chr1, 168750kb	chr18, 4750kb	chr8, 135750kb
**234**	chr11, 69250kb	chr7, 83250kb	chr2, 136750kb	chr19, 46750kb	chr6, 34750kb
**235**	chr18, 46250kb	chr1, 231250kb	chr1, 198750kb	chr8, 97250kb	chr6, 128250kb
**236**	chr18, 67750kb	chr10, 102250kb	chr16, 87750kb	chr9, 16750kb	chr3, 103750kb
**237**	chr1, 114250kb	chr16, 75250kb	chr6, 250kb	chr4, 3250kb	chr18, 67750kb
**238**	chr3, 33250kb	chr9, 34250kb	chr1, 55250kb	chr8, 118750kb	chr5, 150250kb
**239**	chr8, 4250kb	chr14, 67750kb	chr2, 67750kb	chr1, 71250kb	chr4, 103250kb
**240**	chr1, 168750kb	chr1, 55250kb	chr4, 100250kb	chr8, 135750kb	chr10, 125750kb
**241**	chr9, 5250kb	chr9, 128750kb	chr2, 127250kb	chr16, 4250kb	chr5, 96250kb
**242**	chr4, 48250kb	chr1, 68250kb	chr16, 4750kb	chr4, 102750kb	chr12, 40250kb
**243**	chr11, 116250kb	chr2, 181750kb	chr6, 137750kb	chr16, 79750kb	chr6, 137750kb
**244**	chr12, 98250kb	chr10, 90250kb	chr6, 160250kb	chr20, 12250kb	chr10, 81750kb
**245**	chr1, 92250kb	chr1, 78250kb	chr17, 68250kb	chr9, 115750kb	chr1, 36750kb
**246**	chr16, 4750kb	chr17, 40750kb	chr12, 113250kb	chr2, 232750kb	chr6, 151750kb
**247**	chr12, 68250kb	chr7, 99750kb	chr4, 103250kb	chr6, 3250kb	chr10, 90250kb
**248**	chr1, 207750kb	chr11, 69250kb	chr22, 36750kb	chr18, 67750kb	chr21, 43750kb
**249**	chr2, 70250kb	chr6, 139750kb	chr12, 9250kb	chr11, 85750kb	chr6, 3250kb
**250**	chr10, 125750kb	chr18, 67750kb	chr16, 49750kb	chr12, 115250kb	chr3, 25250kb
**251**	chr5, 95250kb	chr3, 33750kb	chr3, 101250kb	chr5, 171750kb	chr4, 102750kb
**252**	chr16, 6250kb	chr6, 34750kb	chr1, 231250kb	chr14, 35750kb	chr5, 95250kb
**253**	chr9, 4250kb	chr7, 149250kb	chr9, 107250kb	chr2, 43750kb	chr2, 62250kb
**254**	chr1, 226750kb	chr2, 203750kb	chr8, 135750kb	chr20, 44750kb	chr3, 119250kb
**255**	chr6, 33250kb	chr2, 163250kb	chr7, 74250kb	chr9, 116250kb	chr3, 167750kb
**256**	chr3, 119250kb	chr11, 118750kb	chr12, 111250kb	chr22, 41250kb	chr18, 46250kb
**257**	chr4, 38250kb	chr12, 9250kb	chr1, 110250kb	chr1, 35250kb	chr5, 79750kb
**258**	chr5, 159750kb	chr8, 113250kb	chr2, 67250kb	chr17, 32750kb	chr8, 10750kb
**259**	chr2, 127250kb	chr4, 3250kb	chr12, 57750kb	chr5, 95250kb	chr12, 111250kb
**260**	chr16, 10750kb	chr6, 3250kb	chr9, 128750kb	chr2, 174750kb	chr1, 114250kb
**261**	chr10, 114750kb	chr5, 111250kb	chr1, 155250kb	chr1, 68250kb	chr5, 10750kb
**262**	chr19, 1250kb	chr8, 23250kb	chr1, 161250kb	chr20, 10750kb	chr1, 168750kb
**263**	chr16, 79750kb	chr7, 74250kb	chr5, 137750kb	chr6, 139750kb	chr2, 136750kb
**264**	chr4, 102750kb	chr11, 10750kb	chr6, 139750kb	chr6, 138250kb	chr5, 111250kb

Order	LR weight	LR PFI	LGBM gain	LGBM PFI	ResDN3 PFI
**265**	chr8, 129250kb	chr7, 123250kb	chr16, 79750kb	chr7, 83250kb	chr10, 60250kb
**266**	chr1, 55250kb	chr2, 204750kb	chr7, 56750kb	chr15, 93250kb	chr19, 46250kb
**267**	chr2, 98250kb	chr13, 107750kb	chr11, 63750kb	chr13, 43250kb	chr10, 102250kb
**268**	chr17, 46250kb	chr3, 33250kb	chr5, 72250kb	chr8, 57250kb	chr20, 23750kb
**269**	chr19, 18750kb	chr12, 56750kb	chr14, 30750kb	chr3, 33750kb	chr5, 750kb
**270**	chr6, 34250kb	chr3, 50750kb	chr6, 33250kb	chr2, 241750kb	chr2, 98250kb
**271**	chr18, 39750kb	chr20, 47250kb	chr21, 40250kb	chr16, 86250kb	chr18, 48250kb
**272**	chr2, 232750kb	chr6, 20250kb	chr16, 10750kb	chr4, 26250kb	chr10, 6750kb
**273**	chr2, 43750kb	chr9, 116250kb	chr14, 88250kb	chr2, 62750kb	chr4, 77750kb
**274**	chr8, 23250kb	chr8, 129250kb	chr7, 102250kb	chr2, 219250kb	chr16, 6250kb
**275**	chr1, 155250kb	chr7, 81750kb	chr2, 182750kb	chr14, 32250kb	chr2, 237750kb
**276**	chr10, 81750kb	chr6, 29250kb	chr19, 55250kb	chr21, 40250kb	chr8, 23250kb
**277**	chr14, 54250kb	chr6, 105750kb	chr1, 25250kb	chr5, 105750kb	chr2, 43250kb
**278**	chr8, 57250kb	chr7, 56750kb	chr15, 50250kb	chr6, 160250kb	chr2, 219250kb
**279**	chr4, 77750kb	chr19, 46250kb	chr1, 151750kb	chr9, 113750kb	chr5, 176750kb
**280**	chr5, 96250kb	chr5, 35750kb	chr4, 187250kb	chr13, 40250kb	chr3, 50250kb
**281**	chr5, 111250kb	chr2, 98250kb	chr2, 33250kb	chr7, 128750kb	chr5, 72750kb
**282**	chr5, 75750kb	chr1, 7750kb	chr18, 39750kb	chr5, 72250kb	chr11, 33750kb
**283**	chr18, 72250kb	chr14, 69250kb	chr7, 20250kb	chr12, 111250kb	chr18, 39750kb
**284**	chr14, 67750kb	chr12, 107250kb	chr2, 22250kb	chr19, 55250kb	chr17, 75750kb
**285**	chr12, 56750kb	chr4, 77750kb	chr18, 33250kb	chr11, 16250kb	chr2, 203750kb
**286**	chr17, 72750kb	chr3, 167750kb	chr1, 67250kb	chr16, 31250kb	chr6, 20250kb
**287**	chr6, 128250kb	chr2, 182750kb	chr20, 12250kb	chr1, 155250kb	chr16, 31250kb
**288**	chr1, 2250kb	chr7, 20250kb	chr7, 28250kb	chr1, 92250kb	chr19, 33250kb
**289**	chr1, 110250kb	chr12, 56250kb	chr2, 236750kb	chr6, 165750kb	chr14, 67750kb
**290**	chr2, 24750kb	chr1, 207750kb	chr12, 68750kb	chr17, 68250kb	chr19, 57250kb
**291**	chr14, 69250kb	chr9, 115750kb	chr21, 45750kb	chr2, 182750kb	chr13, 44750kb
**292**	chr18, 48250kb	chr1, 2250kb	chr10, 64750kb	chr2, 97250kb	chr7, 107250kb
**293**	chr1, 231250kb	chr6, 127750kb	chr2, 203750kb	chr14, 69250kb	chr5, 141250kb
**294**	chr2, 46250kb	chr8, 130750kb	chr7, 81750kb	chr20, 47250kb	chr17, 72750kb
**295**	chr22, 45250kb	chr11, 85750kb	chr4, 115250kb	chr3, 48750kb	chr11, 15750kb
**296**	chr5, 87750kb	chr3, 103750kb	chr1, 185250kb	chr3, 10250kb	chr8, 113250kb
**297**	chr3, 38750kb	chr15, 93250kb	chr8, 4250kb	chr16, 10750kb	chr3, 33750kb
**298**	chr14, 81250kb	chr9, 135750kb	chr1, 22250kb	chr19, 54750kb	chr14, 88250kb
**299**	chr5, 143250kb	chr3, 46250kb	chr20, 47250kb	chr17, 40750kb	chr6, 33250kb
**300**	chr2, 67750kb	chr13, 44750kb	chr4, 122750kb	chr2, 62250kb	chr2, 86750kb
**301**	chr10, 81250kb	chr5, 137750kb	chr17, 32250kb	chr2, 43250kb	chr11, 115250kb
**302**	chr5, 176750kb	chr13, 24750kb	chr6, 34750kb	chr6, 127750kb	chr12, 9250kb
**303**	chr9, 123750kb	chr19, 55250kb	chr13, 44750kb	chr9, 5250kb	chr2, 162750kb
**304**	chr2, 163250kb	chr7, 27250kb	chr14, 35750kb	chr19, 18750kb	chr18, 20250kb
**305**	chr2, 33250kb	chr6, 6750kb	chr2, 61750kb	chr12, 9250kb	chr1, 207250kb
**306**	chr1, 214750kb	chr1, 214750kb	chr16, 87250kb	chr10, 125750kb	chr10, 80250kb
**307**	chr1, 160750kb	chr16, 79750kb	chr11, 118750kb	chr1, 160250kb	chr14, 55250kb
**308**	chr6, 139750kb	chr3, 113750kb	chr18, 46250kb	chr16, 87750kb	chr8, 130750kb

Order	LR weight	LR PFI	LGBM gain	LGBM PFI	ResDN3 PFI
**309**	chr2, 22250kb	chr2, 24750kb	chr6, 33750kb	chr5, 96250kb	chr13, 43250kb
**310**	chr20, 47250kb	chr10, 125750kb	chr5, 130250kb	chr5, 72750kb	chr6, 159250kb
**311**	chr3, 103750kb	chr16, 4250kb	chr7, 157250kb	chr2, 70250kb	chr2, 103250kb
**312**	chr16, 86250kb	chr1, 155250kb	chr14, 98250kb	chr1, 8250kb	chr2, 102250kb
**313**	chr3, 50750kb	chr4, 122750kb	chr8, 129250kb	chr9, 123250kb	chr5, 35750kb
**314**	chr14, 30750kb	chr1, 71250kb	chr4, 90250kb	chr20, 43250kb	chr16, 86250kb
**315**	chr2, 191250kb	chr6, 34250kb	chr6, 105750kb	chr6, 35250kb	chr16, 87250kb
**316**	chr5, 137750kb	chr13, 53250kb	chr5, 750kb	chr8, 143750kb	chr3, 23250kb
**317**	chr7, 17250kb	chr14, 35250kb	chr3, 33750kb	chr20, 60250kb	chr4, 3250kb
**318**	chr7, 128750kb	chr4, 38250kb	chr18, 67750kb	chr14, 54250kb	chr9, 123250kb
**319**	chr1, 71250kb	chr5, 171750kb	chr19, 10750kb	chr7, 99750kb	chr9, 95250kb
**320**	chr3, 113750kb	chr11, 116250kb	chr9, 113750kb	chr20, 3750kb	chr1, 120250kb
**321**	chr11, 118750kb	chr1, 92250kb	chr6, 165750kb	chr2, 160750kb	chr7, 28250kb
**322**	chr1, 173250kb	chr2, 19250kb	chr6, 43250kb	chr17, 72750kb	chr15, 63250kb
**323**	chr16, 30750kb	chr6, 151750kb	chr6, 127750kb	chr1, 173250kb	chr10, 54750kb
**324**	chr6, 134250kb	chr18, 48250kb	chr14, 55250kb	chr3, 122250kb	chr12, 68250kb
**325**	chr9, 34250kb	chr18, 39750kb	chr3, 122250kb	chr12, 68750kb	chr1, 214750kb
**326**	chr1, 192250kb	chr1, 168750kb	chr16, 86250kb	chr15, 41750kb	chr19, 18750kb
**327**	chr18, 68750kb	chr2, 127250kb	chr1, 12250kb	chr12, 48250kb	chr4, 38250kb
**328**	chr1, 25250kb	chr2, 174750kb	chr11, 16250kb	chr16, 27250kb	chr10, 62250kb
**329**	chr14, 39250kb	chr12, 111250kb	chr8, 118750kb	chr22, 45750kb	chr1, 110250kb
**330**	chr2, 203750kb	chr20, 14250kb	chr4, 8750kb	chr14, 81250kb	chr21, 40250kb
**331**	chr21, 43750kb	chr15, 54750kb	chr10, 31250kb	chr6, 34750kb	chr2, 127250kb
**332**	chr16, 9750kb	chr21, 43750kb	chr1, 120750kb	chr10, 114750kb	chr1, 71250kb
**333**	chr2, 174750kb	chr7, 22750kb	chr12, 115250kb	chr18, 56750kb	chr2, 19250kb
**334**	chr16, 68750kb	chr16, 86250kb	chr5, 143250kb	chr21, 43750kb	chr9, 34750kb
**335**	chr6, 65750kb	chr3, 119250kb	chr9, 16750kb	chr5, 141250kb	chr12, 8750kb
**336**	chr10, 80250kb	chr2, 186250kb	chr8, 23250kb	chr1, 66750kb	chr3, 118250kb
**337**	chr19, 54750kb	chr16, 6250kb	chr4, 190750kb	chr5, 39750kb	chr12, 111750kb
**338**	chr16, 77250kb	chr10, 114750kb	chr2, 211750kb	chr10, 97250kb	chr18, 56750kb
**339**	chr7, 107250kb	chr5, 95250kb	chr2, 219250kb	chr6, 6750kb	chr1, 2250kb
**340**	chr16, 87250kb	chr16, 30750kb	chr5, 105750kb	chr1, 20250kb	chr22, 45250kb
**341**	chr9, 80250kb	chr19, 18750kb	chr1, 205250kb	chr5, 11750kb	chr17, 68250kb
**342**	chr9, 116250kb	chr9, 123750kb	chr21, 40750kb	chr10, 31250kb	chr5, 105750kb
**343**	chr7, 81750kb	chr8, 135750kb	chr16, 9750kb	chr15, 30750kb	chr8, 143750kb
**344**	chr8, 130750kb	chr6, 153750kb	chr12, 48250kb	chr6, 33250kb	chr6, 29250kb
**345**	chr6, 127750kb	chr20, 19750kb	chr3, 171750kb	chr1, 7750kb	chr6, 6750kb
**346**	chr20, 43250kb	chr1, 201250kb	chr5, 72750kb	chr9, 135750kb	chr12, 56750kb
**347**	chr18, 53250kb	chr5, 39750kb	chr12, 58250kb	chr13, 44750kb	chr3, 188750kb
**348**	chr3, 10250kb	chr2, 61750kb	chr7, 128750kb	chr4, 184750kb	chr1, 173250kb
**349**	chr3, 95750kb	chr3, 118250kb	chr18, 21750kb	chr1, 168750kb	chr16, 27250kb
**350**	chr5, 105750kb	chr10, 80250kb	chr8, 97250kb	chr5, 8250kb	chr5, 143250kb
**351**	chr20, 19750kb	chr1, 110250kb	chr19, 18750kb	chr2, 100750kb	chr1, 1750kb
**352**	chr1, 201250kb	chr22, 45250kb	chr18, 53250kb	chr20, 1250kb	chr7, 123250kb

Order	LR weight	LR PFI	LGBM gain	LGBM PFI	ResDN3 PFI
**353**	chr2, 102750kb	chr1, 155750kb	chr9, 115750kb	chr1, 101750kb	chr20, 12250kb
**354**	chr3, 188750kb	chr9, 4250kb	chr2, 237750kb	chr16, 30750kb	chr16, 79750kb
**355**	chr3, 167750kb	chr1, 1750kb	chr1, 114750kb	chr3, 38750kb	chr18, 62250kb
**356**	chr15, 63250kb	chr16, 87250kb	chr18, 72250kb	chr1, 27250kb	chr6, 27250kb
**357**	chr10, 64750kb	chr17, 75750kb	chr4, 55750kb	chr20, 1750kb	chr6, 11250kb
**358**	chr7, 123250kb	chr6, 165750kb	chr1, 38250kb	chr4, 25250kb	chr1, 59250kb
**359**	chr16, 4250kb	chr16, 27250kb	chr16, 6250kb	chr2, 19250kb	chr16, 68250kb
**360**	chr14, 32250kb	chr18, 72250kb	chr11, 4750kb	chr9, 107250kb	chr3, 122250kb
**361**	chr20, 36750kb	chr20, 12250kb	chr14, 92750kb	chr1, 207250kb	chr17, 45750kb
**362**	chr3, 118250kb	chr5, 75750kb	chr6, 65750kb	chr12, 111750kb	chr15, 95750kb
**363**	chr6, 34750kb	chr14, 30750kb	chr17, 45750kb	chr5, 130250kb	chr11, 85750kb
**364**	chr7, 102250kb	chr1, 11750kb	chr5, 111250kb	chr10, 50250kb	chr2, 228750kb
**365**	chr2, 138250kb	chr1, 207250kb	chr5, 35750kb	chr20, 54250kb	chr6, 28250kb
**366**	chr15, 93250kb	chr3, 49250kb	chr4, 38250kb	chr2, 24750kb	chr5, 7750kb
**367**	chr12, 62250kb	chr17, 45750kb	chr5, 9750kb	chr7, 22750kb	chr1, 80750kb
**368**	chr9, 95250kb	chr1, 101750kb	chr4, 123750kb	chr1, 185250kb	chr10, 114750kb
**369**	chr8, 97250kb	chr8, 143750kb	chr11, 117750kb	chr1, 117250kb	chr20, 6250kb
**370**	chr6, 33750kb	chr16, 68750kb	chr20, 10750kb	chr17, 45750kb	chr6, 105750kb
**371**	chr5, 11750kb	chr1, 36750kb	chr8, 75250kb	chr12, 58250kb	chr10, 59750kb
**372**	chr3, 38250kb	chr5, 750kb	chr5, 96250kb	chr2, 67250kb	chr7, 99750kb
**373**	chr15, 26750kb	chr12, 98250kb	chr1, 11750kb	chr5, 750kb	chr2, 185750kb
**374**	chr9, 75250kb	chr1, 17750kb	chr6, 23250kb	chr5, 137750kb	chr11, 116250kb
**375**	chr8, 135750kb	chr9, 123250kb	chr14, 39250kb	chr2, 163250kb	chr6, 127750kb
**376**	chr2, 61750kb	chr3, 188750kb	chr5, 87750kb	chr20, 23750kb	chr11, 118750kb
**377**	chr6, 160250kb	chr3, 122250kb	chr2, 70250kb	chr6, 34250kb	chr21, 40750kb
**378**	chr2, 228750kb	chr8, 57250kb	chr1, 212250kb	chr15, 90250kb	chr9, 21750kb
**379**	chr5, 8250kb	chr10, 62250kb	chr4, 80750kb	chr1, 25250kb	chr1, 68250kb
**380**	chr5, 45250kb	chr16, 87750kb	chr1, 66750kb	chr10, 62250kb	chr20, 38250kb
**381**	chr5, 79750kb	chr12, 101750kb	chr2, 97250kb	chr12, 133250kb	chr12, 16750kb
**382**	chr2, 56250kb	chr6, 33750kb	chr11, 10750kb	chr18, 46250kb	chr6, 90750kb
**383**	chr5, 171750kb	chr2, 70250kb	chr6, 169750kb	chr6, 27250kb	chr20, 48250kb
**384**	chr21, 40250kb	chr11, 103250kb	chr10, 44750kb	chr18, 72250kb	chr4, 184750kb
**385**	chr4, 122250kb	chr13, 113750kb	chr6, 20250kb	chr2, 125750kb	chr6, 153750kb
**386**	chr15, 37750kb	chr6, 170250kb	chr15, 95750kb	chr6, 20250kb	chr20, 53750kb
**387**	chr20, 6250kb	chr17, 72750kb	chr3, 103750kb	chr5, 35750kb	chr7, 121750kb
**388**	chr19, 10750kb	chr2, 191750kb	chr20, 19750kb	chr6, 158750kb	chr16, 87750kb
**389**	chr14, 89750kb	chr12, 16750kb	chr9, 135750kb	chr6, 29250kb	chr1, 92250kb
**390**	chr1, 11750kb	chr9, 34750kb	chr12, 8750kb	chr7, 4750kb	chr19, 48750kb
**391**	chr11, 85750kb	chr5, 105750kb	chr2, 62250kb	chr1, 214750kb	chr5, 75750kb
**392**	chr7, 148750kb	chr1, 66750kb	chr3, 39750kb	chr6, 16250kb	chr14, 30750kb
**393**	chr5, 35750kb	chr7, 157250kb	chr16, 78250kb	chr10, 64750kb	chr4, 55750kb
**394**	chr20, 53750kb	chr10, 31250kb	chr18, 68750kb	chr5, 75750kb	chr1, 101750kb
**395**	chr1, 152750kb	chr2, 228750kb	chr10, 81750kb	chr7, 136250kb	chr12, 98250kb
**396**	chr11, 16250kb	chr14, 55250kb	chr5, 158250kb	chr7, 56750kb	chr4, 122250kb

Order	LR weight	LR PFI	LGBM gain	LGBM PFI	ResDN3 PFI
**397**	chr17, 29250kb	chr20, 10750kb	chr2, 220750kb	chr2, 229250kb	chr2, 163250kb
**398**	chr3, 171750kb	chr8, 58750kb	chr3, 10250kb	chr3, 159750kb	chr20, 43250kb
**399**	chr13, 68750kb	chr19, 10750kb	chr3, 119250kb	chr2, 61750kb	chr9, 20250kb
**400**	chr10, 72750kb	chr9, 95250kb	chr2, 228750kb	chr12, 2250kb	chr3, 194250kb

Table S1. Lists of the 400 most important loci.
We list here the list of the 400 most important loci selected, when averaged over 10 different folds of the dataset, respectively by Lasso regularization with weight criterion (LR weight), Lasso regularization with permutation feature importance criterion (LR PFI), LightGBM with gain criterion (LGBM gain), LightGBM with permutation feature importance criterion (LGBM PFI), and a dense residual neural network with 3 hidden layers with permutation feature importance criterion (ResDN3 PFI). For each locus, we indicate the chromosome and the center of the corresponding 500kb window (250kb on each side), in hg19 coordinates.

References

[1] Pedregosa F, Varoquaux G, Gramfort A, Michel V, Thirion B, Grisel O, et al. Scikit-learn: Machine Learning in Python. Journal of Machine Learning Research. 2011;12:2825–2830.

[2] Chollet F, et al. Keras; 2015. https://keras.io.

[3] Abadi M, Agarwal A, Barham P, Brevdo E, Chen Z, Citro C, et al. TensorFlow: Large-Scale Machine Learning on Heterogeneous Systems; 2015. Available from: https://www.tensorflow.org/.

[4] Chen T, Guestrin C. Xgboost: A scalable tree boosting system. In: Proceedings of the 22nd acm sigkdd international conference on knowledge discovery and data mining. ACM; 2016. p. 785–794.

[5] Ke G, Meng Q, Finley T, Wang T, Chen W, Ma W, et al. LightGBM: A highly efficient gradient boosting decision tree. In: Advances in Neural Information Processing Systems; 2017. p. 3149–3157.

[6] Prokhorenkova L, Gusev G, Vorobev A, Veronika Dorogush A, Gulin A. CatBoost: unbiased boosting with categorical features. arXiv preprint arXiv:170609516. 2017.
